# Regulation of epithelial integrity and organ growth by Tctp and Coracle in *Drosophila*

**DOI:** 10.1371/journal.pgen.1008885

**Published:** 2020-06-19

**Authors:** Sung-Ryeong Lee, Sung-Tae Hong, Kwang-Wook Choi

**Affiliations:** 1 Department of Biological Sciences, Korea Advanced Institute of Science and Technology, Daejeon, Republic of Korea; 2 Department of Anatomy & Cell Biology, Department of Medical Science, College of Medicine, Chungnam National University, Daejeon, Republic of Korea; HudsonAlpha Institute for Biotechnology, UNITED STATES

## Abstract

Regulation of cell junctions is crucial for the integrity of epithelial tissues and organs. Cell junctions also play roles in controlling cell proliferation for organ growth. Translationally controlled tumor protein (TCTP) is a conserved protein involved in growth control, but its role in cell junctions is unknown. Here we show that *Drosophila* Tctp directly interacts with the septate junction protein Coracle (Cora) to regulate epithelial integrity and organ growth. Tctp localizes together with Cora in the epidermis of the embryo. Loss of Cora reduces the level of Tctp in the epidermis but not *vice versa*. *cora/+* or *Tctp/+* single heterozygotes develop normally to adulthood. However, double heterozygotes for *cora* and *Tctp* mutations show severe disruption of epithelia causing synthetic lethality in the embryo. Double knockdown of Cora and Tctp in eye imaginal disc synergistically leads to disruption of the eye disc, resulting in a severe reduction or loss of eye and head. Conversely, double knockdown of Cora and Tctp in wing disc causes overgrowth as well as cell death. Inhibition of cell death under this condition causes hyperplastic growth of the wing disc. Tctp also shows direct and functional interaction with Cora-associated factors like Yurt and Na^+^/K^+^-ATPase. This study suggests that proper levels of Tctp and Cora are essential for the maintenance of the Cora complex and the integrity of epithelia. Our data also provide evidence that both Cora and Tctp are required to suppress overgrowth in developing wing.

## Author summary

Organ growth depends on intercellular signaling for cell proliferation. Accumulating evidence indicates that tissue growth is also regulated by cell junctions. Translationally controlled tumor protein (TCTP) is an evolutionarily conserved protein family implicated in cancer. In *Drosophila*, Tctp is required for diverse cytoplasmic and nuclear functions including organ growth, DNA repair, and chromatin regulation during development. However, it is unknown whether Tctp plays an additional role in cell junctions at the membrane. Here we show that Tctp localizes together with the FERM domain protein Coracle (Cora) at the basolateral septate junctions in some tissues. Our data suggest that Cora is required for the maintenance of Tctp in the cell membrane but not *vice versa*. Tctp forms a complex with Cora, and the mutations in *cora* and *Tctp* genes show synergistic genetic interaction in the embryo and developing organs. Remarkably, the reduction of Cora and Tctp can induce massive overgrowth in the wing. We propose that the direct interaction of Tctp with the Cora junction complex is required for epithelial integrity and organ growth during development.

## Introduction

Growth regulation is crucial for normal development in all animals. The development of animal organs depends on proliferation, cell growth, and cell death. These cellular events are regulated by diverse signaling pathways. Identification of specific factors that induce or mediate growth signaling is key for understanding the mechanisms for growth control. TCTP is a protein family that has emerged as a multifaceted regulator of tissue growth and survival [1, 2, 3]. TCTP has been implicated in cancer based on the finding that it is most differentially expressed between normal and cancer cells [4, 5]. Notably, TCTP is up-regulated in various tumor cells derived from epithelial tissues, while suppression of TCTP can revert malignant tumors to normal cells through a process of tumor reprogramming [6, 7]. Mammalian TCTP is involved in the regulation of cell growth, cell cycle, apoptosis, malignant transformation, and other cellular processes by interacting with diverse proteins, including p53 and BCL [8, 9, 10, 11].

TCTP family proteins are evolutionarily conserved in eukaryotes. *In vivo* function of TCTP in developing animals has been identified in *Drosophila* [12]. Genetic analysis has shown that *Drosophila* TCTP (‘Tctp’ hereafter) is required for controlling both cell number and size during organ development. Tctp is essential for organ growth by regulating the Target of Rapamycin (TOR) signaling [13]. Evidence suggests that TCTP proteins in other species are also involved in TOR signaling [14, 15, 16, 17]. Tctp and its mammalian homologs play additional roles in genome stability by regulating DNA repair [18, 19]. Furthermore, Tctp participates in epigenetic regulation of gene expression by interacting with the chromatin remodeling factor Brahma (Swi/SNF) and is necessary for the maintenance of heterochromatin in pericentromeric regions [20]. These studies indicate that Tctp is widely distributed in the cytoplasm and the nucleus to regulate diverse cellular events, including growth signaling, genome stability, and gene regulation.

In this study, we identified the FERM domain protein Coracle (Cora) as a binding partner of Tctp. Cora is the *Drosophila* homolog of the vertebrate Protein 4.1 family and a core member of the septate junctions of epithelial tissues. The Cora-Tctp binding was unexpected because there has been no known role for Tctp in cell junctions. There is increasing evidence that cell junctions play roles for growth signaling during development [21]. In *Drosophila*, septate junction proteins such as Discs-large (Dlg), Scribble (Scrib), and Lethal giant larvae (Lgl) function together to maintain apicobasal cell polarity in developing epithelia [22, 23]. These proteins are known as tumor suppressors since the loss of any of these proteins results in tumorous growth in imaginal discs and adult organs [24, 25, 26, 27].

Cora forms another septate junction complex with Yurt (Yrt), Neurexin IV (Nrx-IV), and Na^+^/K^+^-exchanging ATPase α subunit (ATPα). The Cora complex proteins cooperatively function to promote the basolateral membrane stability by negatively interacting with the apical determinant Crumbs (Crb) [28]. Among these proteins, Cora is essential for the barrier function of the septate junction and dorsal closure of embryonic epithelia [29, 30]. In imaginal discs, *cora* loss-of-function mutant tissues are defective in growth, suggesting that Cora is required for cell proliferation in contrast to the tumor suppressor function of the Dlg complex. It is unknown how these two protein complexes in septate junctions display opposite functions in organ growth. Our finding of the physical interaction between Cora and Tctp raises an intriguing question of whether Cora and Tctp act together to regulate epithelial integrity and organ development *in vivo*.

In this study, we demonstrate physical and functional interaction between Cora and Tctp. Double heterozygotes for *cora* and *Tctp* mutations show synthetic disruption of embryonic development. Cora is required to maintain the level of Tctp. We show that proper levels of Cora and Tctp are required for the growth of eye imaginal disc. In contrast, loss of Cora and Tctp results in hyperplastic growth in the wing disc. We propose that Tctp is required to maintain the integrity of epithelia through multiple interactions with Cora complex proteins.

## Results

### Cora interacts with Tctp with overlapping localization in epidermal epithelia of embryo

**Fig 1:**
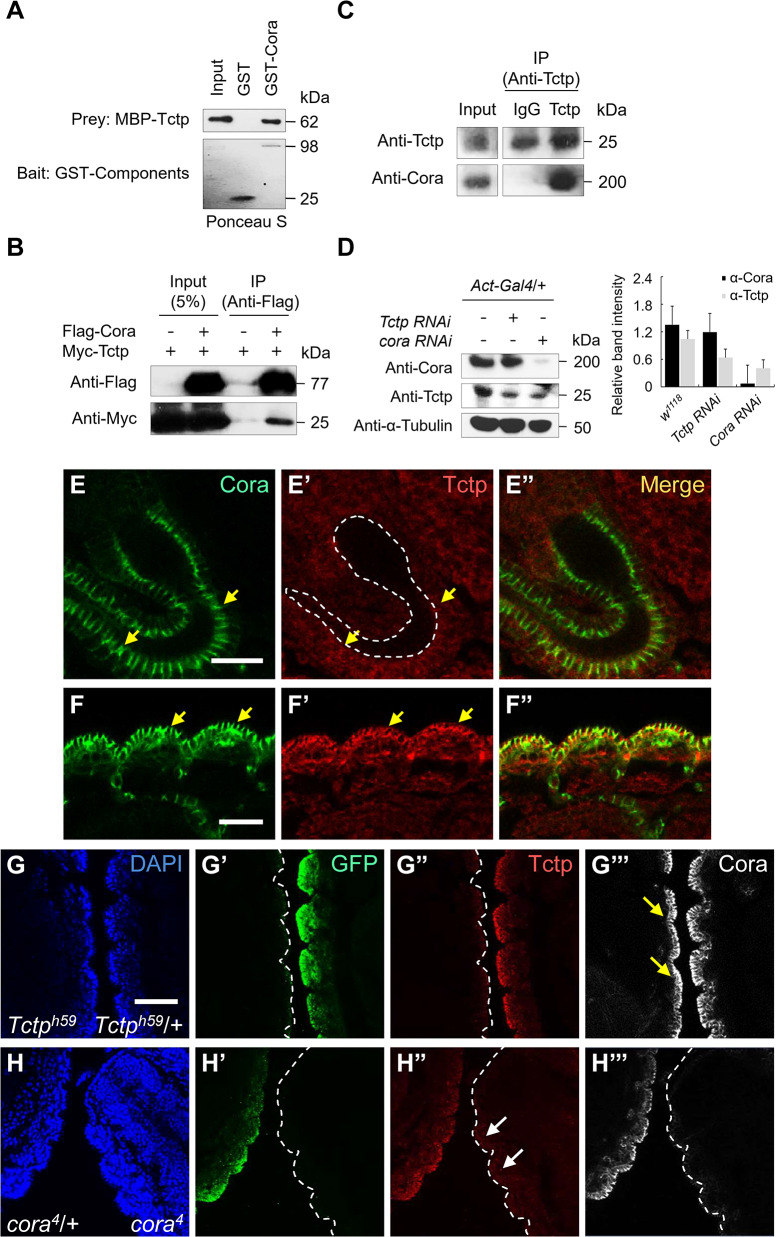
Cora interacts with Tctp and is required for Tctp maintenance. (A) GST-pull down of Cora and Tctp. GST-Cora directly binds to MBP-Tctp. The upper blot shows MBP-Tctp pulled down by GST-Cora and stained with an anti-MBP antibody. The lower blot shows GST tagged proteins used for GST-pulldown. (B) Co-immunoprecipitation of Cora and Tctp using S2 cells. 5% input was used as a positive control for western blotting. Myc-Tctp is co-immunoprecipitated by Flag-Cora. (C) An endogenous Cora isoform of about 200 kDa is co-immunoprecipitated with Tctp from wild-type embryos. (D) Western blot analysis of Tctp and Cora levels in the embryo. *Tctp RNAi* using *actin*-*Gal4* does not affect the Cora protein level. In contrast, *cora RNAi* reduces the level of Tctp. The graph shows the quantification of three western blot results. (E-F”) Embryos stained for Cora and Tctp. In hindgut, Cora (green) is localized to the basolateral membrane (E, arrows) while Tctp (red) is ubiquitously distributed (E’). Dashed lines indicate the position of the lumen of the hindgut. In the epidermis, Cora and Tctp show overlapping localization at the basolateral membrane (F-F”, arrows). (G-H”) Effects of *cora* or *Tctp* mutation on the level of Cora and Tctp, respectively. *cora*^*4*^ and *Tctp*^*h59*^ were balanced with *CyO*-*GFP* and *TM3*-*GFP*, respectively, to identify the genotypes of progeny. Distribution of Cora (shown as white staining) is normal in *Tctp*^*h59*^ mutant embryo (G”’, arrows). Tctp (shown in red) is strongly reduced in *cora*^*4*^ mutant embryo (H”) at stage 14. The embryonic stage was determined based on the segmented pattern of the epidermis and the shape of the midgut (see S1C–S1D”’ Fig). GFP-positive embryos are heterozygotes for *Tctp*^*h59*^ (G) or *cora*^*4*^ (H). Scale bars, 50 μm.

We performed a phage display library screen to identify new binding partners for Tctp. From a screen of an aptamer library, we found Tctp-binding heptamer peptides containing the YKGPTQV sequence. One of the *Drosophila* proteins containing similar peptide sequences was Cora, a septate junction protein with a FERM domain. The heptamer-matching region of Cora protein (YKGRTQ) showed a 71% amino-acid (AA) identity with the heptamer according to the BLASTP programs. To confirm the direct interaction between Cora and Tctp, we performed GST-pulldown assays. Bacterially expressed GST-Cora could bind to MBP-Tctp fusion protein (Fig 1A). Immunoprecipitation (IP) in S2 cells expressing Flag-Cora and Myc-Tctp also indicated that these proteins form a complex in culture cells (Fig 1B). Furthermore, endogenous Cora and Tctp were co-immunoprecipitated in embryo extracts (Fig 1C), suggesting that they are associated *in vivo*. We used the Gal4-UAS system [31] for knockdown of these proteins by double-stranded RNA (dsRNA) interference (RNAi). Western blot analysis of embryo extracts revealed that the level of Tctp is reduced by knockdown of Cora using *actin*-*Gal4* driver. Conversely, *Tctp RNAi* did not change the level of Cora (Fig 1D). This indicates that Cora is required for maintaining the normal level of Tctp in the embryo.

Physical interaction between Cora and Tctp suggests that these two proteins might co-localize in some tissues. In the embryo, Cora is localized to the septate junctions along the basolateral region of epithelial cell membranes of the epidermis and internal organs [32] (Fig 1E–1E” and 1F–1F”). Tctp is ubiquitously expressed in most tissues of embryo, including hindgut, while Cora is specifically localized to the basolateral membrane of the hindgut (Fig 1E). In the epidermis, however, Tctp is enriched at the membranes of epidermal cells together with Cora (Fig 1F–1F”). To test whether Cora and Tctp are required for their maintenance in the epidermis, we examined their localization in *cora* or *Tctp* mutant background at stage 14 of embryogenesis (Fig 1G–1H”’, S1C–S1D”’ Fig). Cora localization was not significantly changed in *Tctp* null mutant (*Tctp*^*h59*^) embryo (Fig 1G”’). However, the level of Tctp was decreased in *cora*^*4*^ strong hypomorph mutant embryo (Fig 1H”). These data suggest that Cora is required for maintaining Tctp in the epidermal cell membrane.

### Mutations in *cora* and *Tctp* cause synthetic lethality in embryo

**Fig 2:**
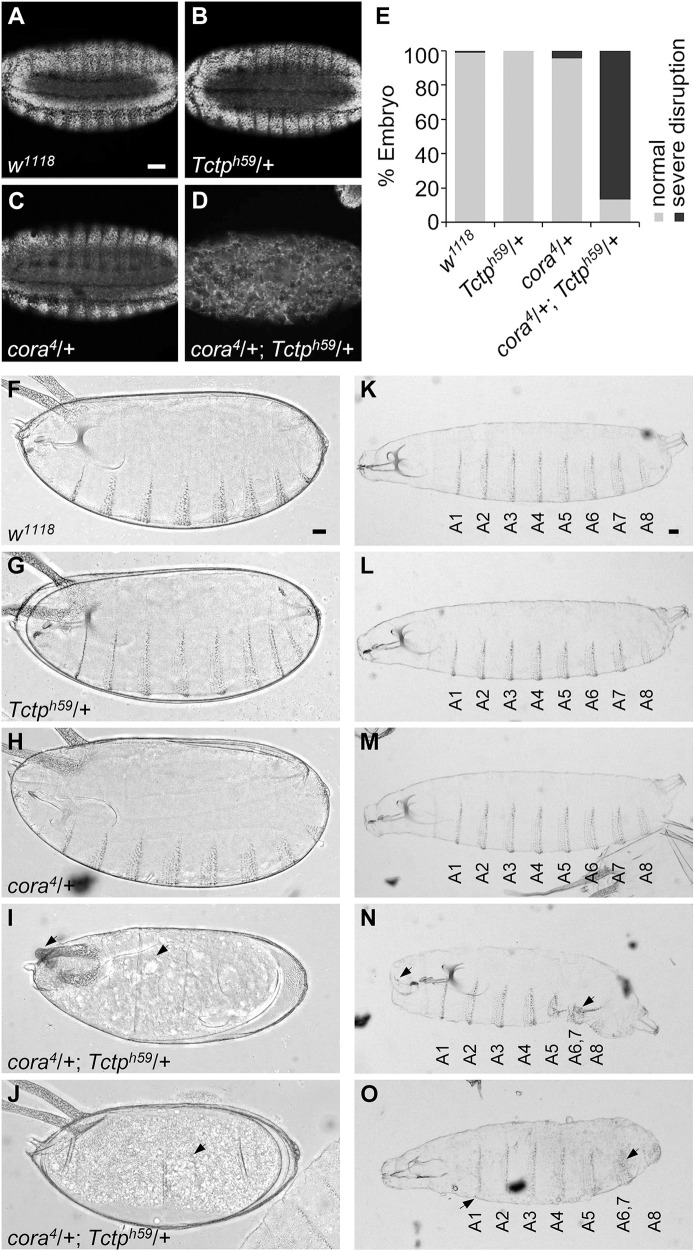
*cora* and *Tctp* heterozygotes show synthetic lethality in the embryo. (A-D) Optical cross-section views of the DAPI staining pattern. Embryo genotypes are as indicated. *Tctp*^*h59*^*/+* (B) or *cora*^*4*^*/+* (C) heterozygotes show relatively normal pattern. Double heterozygous embryos are grossly disorganized (D). (E) Quantification of embryos showing normal morphology and abnormalities. Wild-type and single heterozygous embryos are mostly normal. In contrast, about 87% of double heterozygous embryos show embryo lethality with severe disruption of epithelia. All embryos are at stage 16 (*n ≥* 50 for each genotype). (F-O) Denticle phenotypes in late-stage embryos (F-J) and 1^st^ instar larvae (K-O). Wild-type embryo and larva have normal denticles (F, K). Heterozygotes of *Tctp*^*h59*^*/+* (G, L) or *cora*^*4*^*/+* (H, M) show a normal pattern of denticles. *cora/+; Tctp/+* double heterozygotes die before denticle formation (I, J, arrows). The only survived larvae show fused or decreased number of denticle belts in the posterior segments (N, O, arrows). Scale bars, 50 μm.

To further examine the functional relationship between Cora and Tctp, we tested whether mutations in these genes show any genetic interaction. *Tctp*^*h59*^*/+* or *cora*^*4*^*/+* heterozygous embryos stained with DAPI at stage 16 showed relatively normal segmental body pattern compared with wild-type control (Fig 2A–2C). In striking contrast, 87% (*n* = 45) of double heterozygotes (*cora*^*4*^*/+; Tctp*^*h59*^*/+*) showed embryonic lethality. Nearly all of these dying embryos showed severe disruption of the epidermis and internal structures, resulting in unidentifiable tissue debris (Fig 2D and 2E). We also checked for additional epidermal defects by examining embryo cuticles. Wild-type embryos secrete cuticles near the end of embryogenesis, resulting in the formation of segmentally repeated denticle belts on the ventral epidermis of embryos (Fig 2F) and 1^st^ instar larvae (Fig 2K). *cora*^*4*^*/+* or *Tctp*^*h59*^*/+* heterozygote embryos and larvae showed a normal pattern of denticles (Fig 2G, 2H, 2L and 2M). On the contrary, double heterozygote embryos that fail to hatch did not show obvious signs of denticle formation other than some cuticle debris (29/110, 26%; close to 25% expected double heterozygotes) (Fig 2I and 2J). Occasionally, these embryos showed defective dorsal appendages (Fig 2I). We found rare escaper double heterozygotes that died as first instar larvae (2 out of 29 dead embryos). These larvae showed a loss or fusion of a couple of posterior abdominal segments (Fig 2N and 2O). These data suggest that most double heterozygote embryos died before denticle formation and that escapers are defective in the segmentation of the posterior region. Strong phenotypes of double heterozygous embryos indicate that dosage-sensitive interaction between Cora and Tctp is crucial for the maintenance of epithelia and embryonic development.

### Defective embryogenesis after cellularization in *cora*/*+*; *Tctp*/+ double heterozygotes

**Fig 3:**
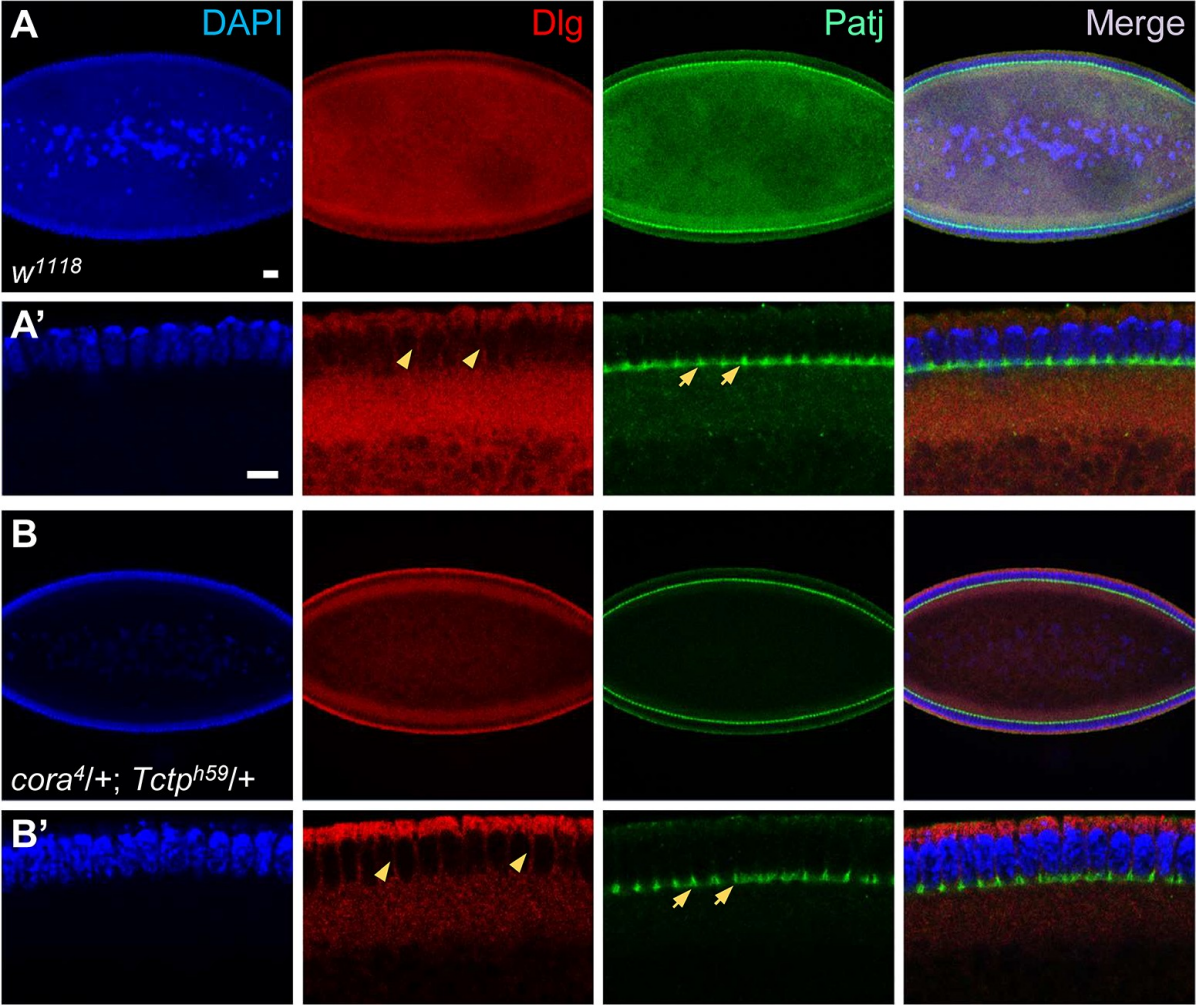
*Cora/+; Tctp/+* double heterozygous embryos show normal cellularization. (A-B’) Cellularizing embryos stained with antibodies for Dlg and Patj. Cellularization of wild-type embryos (A). Enlarged cross section of epidermis shown in (A) (A’). Dlg is localized in the apical and the lateral plasma membrane (PM). Patj is localized in the furrow canals (arrows) basal to the lateral PM (arrowheads). Double heterozygotes show stronger Dlg staining in yolk and cortex density but show no apparent defect in the pattern of cellularization (B). Enlarged cross section of epidermis shown in (B) (B’). Scale bars, 20 μm.

Because *cora*^*4*^*/+; Tctp*^*h59*^/+ double heterozygous embryos showed severe disruption at stage 16, we examined earlier stages to identify possible causes for the defects in late stages of embryogenesis. First, we checked whether Cora and Tctp affect the process of cellularization at stage 5 after the syncytial blastoderm stage. Cellularization begins at the boundaries between somatic buds above nuclei. Furrow canals are induced between the buds [33]. Dlg and Patj are initially concentrated at the furrow canals. As the lateral plasma membrane (PM) extends basally, Patj marks the basal tip whereas Dlg labels the lateral PM (Fig 3A and 3A’). This polarized distribution of junctional proteins persists throughout the cellularization process [34]. *cora*^*4*^*/+; Tctp*^*h59*^/+ double heterozygotes showed decreased Dlg levels in the yolk region but no obvious defects in the pattern of Dlg and Patj localization (Fig 3B and 3B’), suggesting that cellularization occurs normally in double heterozygous embryos.

**Fig 4:**
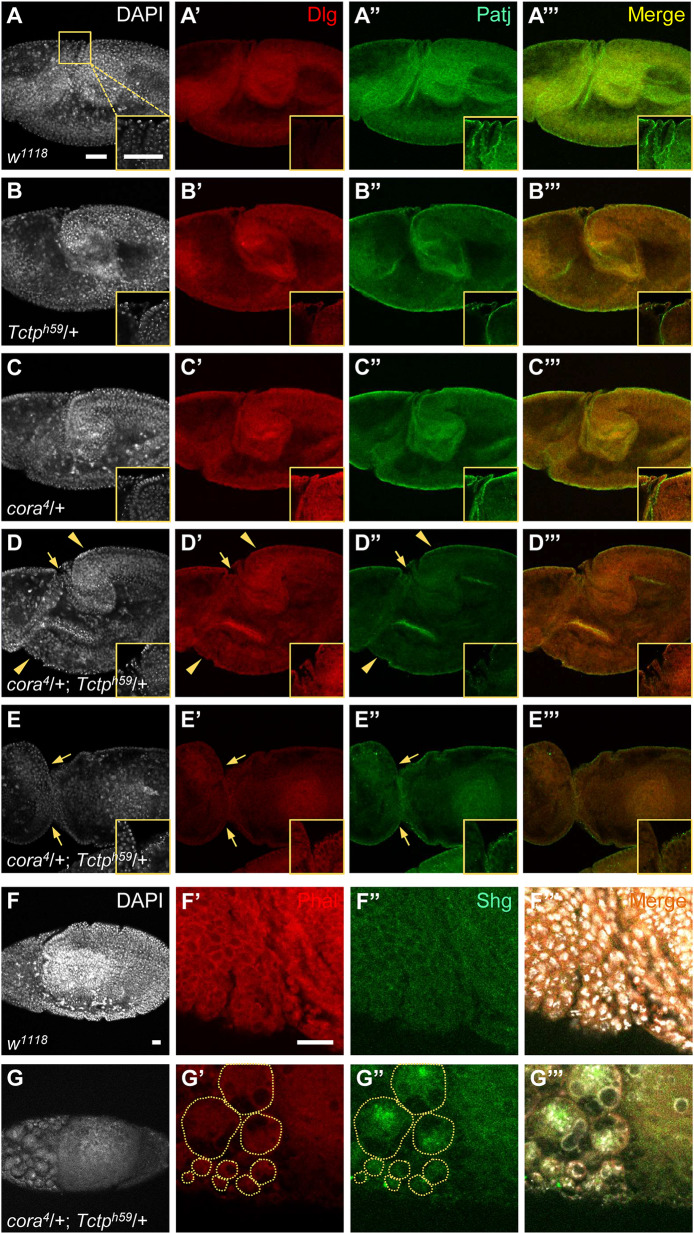
*Cora/+; Tctp/+* double heterozygotes show severe disruption after cellularization. (A-E”’) Cross-sections of embryos stained for Dlg (red) and Patj (green) during germ-band extension in stage 9. The insets at the right bottom corners show enlarged views of the boxed region near the cephalic furrow. Localization of Dlg and Patj in wild-type embryos (A-A”’). At this stage, the expression level of Dlg is low, but high levels of Patj are localized at the apical region. Single heterozygotes *Tctp/+* (B-B”’) and *cora/+* (C-C”’) show reduced Patj levels in the germ-band region. Two examples of double heterozygotes with mild and severe phenotypes. An embryo with mild phenotypes (D-D”’) shows bulging of germband and damaged epithelium near the cephalic furrow with strong reduction in Patj staining. An embryo with severe phenotypes (E-E”’) shows near separation of the cephalic region and the posterior regions. Scale bars, 50 μm. (F-G) F-actin (red) and E-cadherin (green) localization in wild-type embryos during stage 11. Wild-type embryo is normal (F-F”’). *cora/+;Tctp/+* double heterozygotes show severe disruption in the anterior region with diffused DAPI staining (circles, G-G”’). Scale bars, 20 μm.

However, significant abnormalities were found in double heterozygous embryos during germ-band extension at stage 9. Wild-type embryos at this stage showed smooth invagination of the posterior midgut and the cephalic furrow stained by Patj and Dlg antibodies. Single heterozygotes (*cora*^*4*^*/+* or *Tctp*^*h59*^/+) were relatively normal compared with wild-type (Fig 4A–4C”’). In contrast, double heterozygous embryos showed various abnormalities, including bulged germ-band, epidermal defects in the areas of posterior midgut invagination and cephalic furrow (Fig 4D–4D”’). In more severe cases, the cephalic and posterior regions were nearly separated (Fig 4E–4E”’). It was also noted that Patj staining was considerably weakened near the region of cephalic furrow (Fig 4D” and 4E”, insets). Double heterozygous embryos showed severe disruption of embryos in later stages (stage 11), leading to disintegration of nuclei and abnormal aggregation of Shotgun (Shg)/E-cad, as shown in Fig 4G and 4G”.

**Fig 5:**
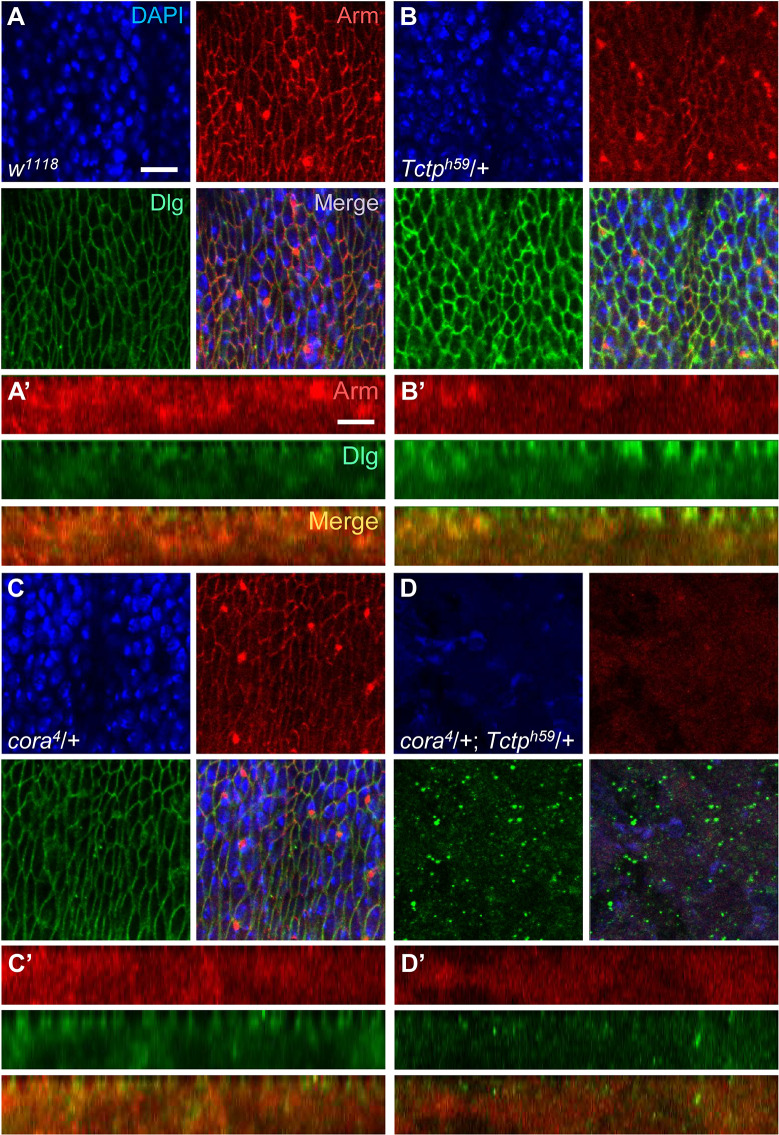
*cora and Tctp* heterozygotes show synergistic genetic interaction in embryo. (A-D’) Tangential sections of embryos stained for DAPI, Arm, and Dlg. Prime (‘) columns are enlarged cross-section views of embryos. Wild-type (*w*^*1118*^) embryo shows normal Arm (red) and Dlg (green) localization in the epidermal epithelium. DAPI staining is shown in blue (A, A’). *Tctp*^*h59*^*/+* heterozygotes show reduced Arm but increased Dlg staining (B, B’). *cora*^*4*^*/+* null heterozygotes show a mild reduction in Arm staining (C, C’). *cora*^*4*^*/+; Tctp*^*h59*^/+ double heterozygotes show severe epidermal disruption in dying embryo (D, D’). All embryos are at stage 16 except that double heterozygotes are arrested earlier. (*n ≥* 50). Scale bars, 10 μm.

We also examined the epithelial integrity by checking the adherens junction marker Armadillo (Arm). *Tctp*^*h59*^*/+* heterozygous embryos at stage 16 showed stronger Dlg and weaker Arm staining than the *w*^*1118*^ wild-type control (Fig 5B and 5B’). *cora*^*4*^*/+* heterozygous embryos also showed slightly weaker Arm staining than wild-type (Fig 5C and 5C’). Despite these differences in the level of staining, almost all *cora*^*4*^*/+* or *Tctp*^*h59*^*/+* heterozygous embryos showed relatively normal cell shape comparable to the wild-type pattern and developed normally to adulthood. In striking contrast, nearly all double heterozygous embryos (87%, n = 45) showed severe disruption of the epidermis and internal structures, resulting in unidentifiable tissue debris (Fig 5D and 5D’). For a better comparison of apical and basolateral pattern, we examined cross-section images of less severe double heterozygote embryos (13%) stained for Dlg and Patj. Compared with the wild-type and single heterozygote control embryos at stage 16 (S2A–S2C”’ Fig), double heterozygotes showed stronger and broader Dlg staining while apical Patj staining is considerably reduced (S2D–S2D”’ Fig), suggesting defects in the apical-basal pattern.

Next, we tested additional double heterozygotes with another strong allele *cora*^*2*^ and a deficiency *Df(2R)Exel6069 (56B5-56C11)* (*Df* in short) uncovering the *cora* gene. Both of these double heterozygotes (*cora*^*2*^*/+; Tctp*^*h59*^*/+* and *Df/+; Tctp*^*h59*^*/+*) showed similar embryo lethal phenotypes (S3D–S3D”’ and S3F–S3F”’ Fig), although synthetic lethal frequencies with *cora*^*2*^ (about 40%, *n* = 41) and *Df* (about 40%, *n* = 58) were lower than that of *cora*^*4*^ (87%) (S3G Fig). *cora*^*2*^ may be weaker than *cora*^*4*^ because it does not affect the essential FERM domain that is partially deleted in *cora*^*4*^ [35]. A lower lethal frequency with *Df* may be due to a different genetic background with a large deletion in the *Df* chromosome.

### Knockdown of Cora and Tctp synergistically disrupts wing development

Tctp is known to be essential for the growth of larval imaginal discs [12]. Cora is also required for cell proliferation in developing discs [30]. To test whether Cora-Tctp interaction is crucial for the growth of adult organs, we examined wings in developing larvae and adult flies. Immunostaining of wild-type wing discs from late third instar larvae showed expression of Cora and Tctp proteins in all disc cells. Cora and Tctp showed similar membrane localization in the hinge region of wing disc (S4A Fig). In the wing pouch, Tctp was broadly expressed in the cytoplasm but was also detected at the cell membrane in a similar pattern as Cora (S4A’ Fig). To address the functional relationship between Cora and Tctp, we tested whether these two genes show genetic interaction in wing development. Since double heterozygotes (*cora*^*4*^*/+; Tctp*^*h59*^*/+*) die during embryogenesis (Figs 2D and 5D), we firstly tested genetic interaction between *cora* and *Tctp*, using *patched (ptc)-Gal4* that drives Gal4 expression along the anterior-posterior boundary between the longitudinal wing veins L3 and L4. Knockdown of Tctp by *ptc-Gal4* (labelled as *ptc*>*Tctp RNAi*) resulted in a reduction of the targeted area between L3 and L4 (S4D Fig), as shown earlier [12]. Unlike *Tctp RNAi*, *cora RNAi* led to embryonic lethality, probably due to *ptc-Gal4* expression in embryonic development. Hence, we utilized the *cora*^*4*^*/+* heterozygous condition that allows nearly normal wing development, although wing size is slightly larger than normal (S4C Fig). When *Tctp RNAi* was combined with *cora*^*4*^*/+* heterozygous mutation, flies could develop to adults but showed significantly smaller wings than *Tctp RNAi* alone (S4E and S4F Fig).

Similar genetic interaction was observed when *cora* and *Tctp RNAi* was induced in the entire wing pouch by using *MS1096-Gal4*. *MS1096*>*Tctp RNAi* resulted in a mild reduction of wing size (S4G Fig). *MS1096*>*cora RNAi* flies survived to adulthood but showed severely reduced and folded wings (S4H Fig). In contrast, the double knockdown of Cora and Tctp by *MS1096-Gal4* led to 100% (*n* = 28) pupal lethality (S4I Fig). These data indicate that Cora and Tctp show strong genetic interaction during wing development.

### Double knockdown of Cora and Tctp causes overgrowth in wing disc

**Fig 6:**
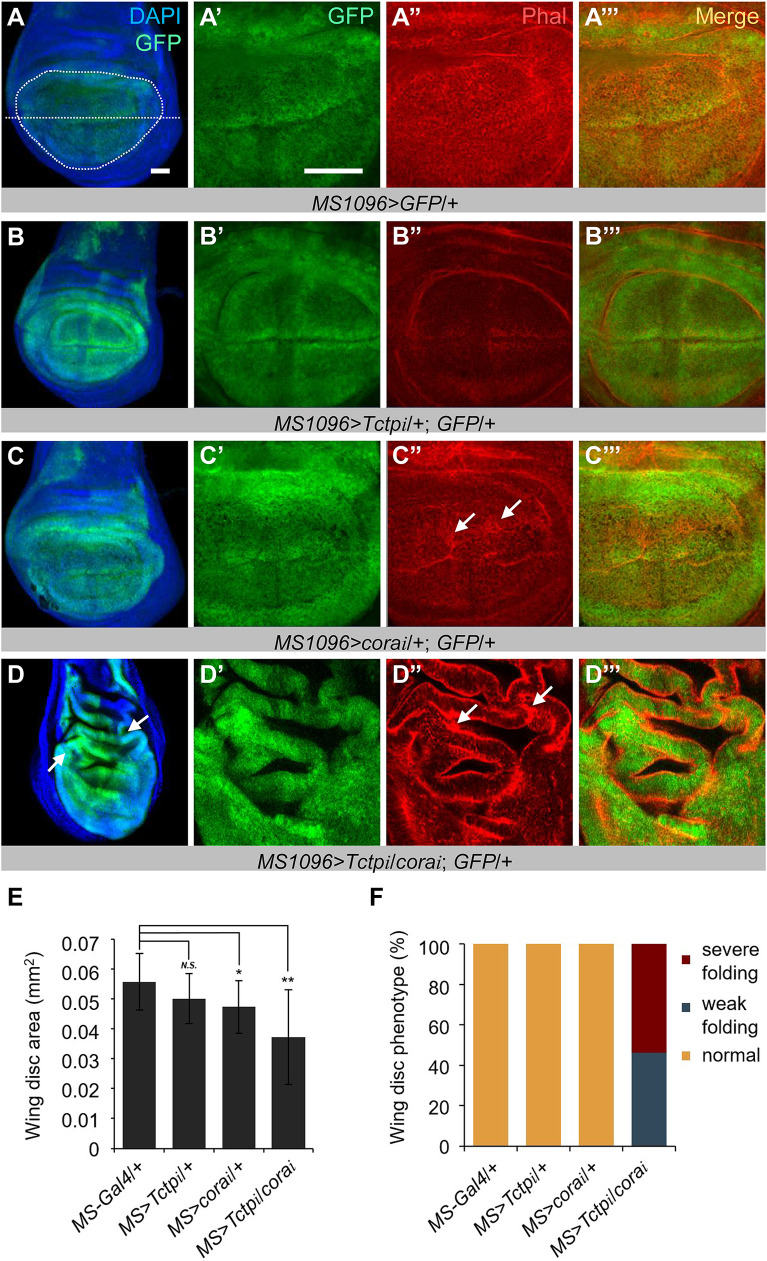
Double knockdown of Cora and Tctp causes abnormal folding in wing disc. (A-D”’) Effects of RNAi by *MS1096*-*Gal4* on wing discs. Wing discs were stained for GFP and Phal. The dotted circle and the horizontal line indicate the wing pouch and the dorsoventral boundary, respectively. *MS1096*-*Gal4* control shows normal wing disc with preferential GFP expression in the wing pouch (A-A”’). *Tctp RNAi* results in slight reduction of the wing disc. Phal staining is also reduced (B-B”’). *cora RNAi* reduces wing disc size. GFP staining in the hinge region is enhanced. These discs show abnormally aggregated Phal staining in some patches of wing tissues (arrows) (C-C”’). Wing discs with double RNAi for Cora and Tctp appear to be smaller than the discs with *cora* or *Tctp* single RNAi. However, most discs show bulging of wrinkled wing pouch. Phal staining is intensely accumulated in the apical or basal part of the wing pouch epithelium (arrows) (D-D”’). (E) Quantification of wing disc size shown in (A-D). (F) Quantification of wing discs with folded wing pouch shown in (A-D). Error bars are s.e.m. (*n ≥* 13). *N*.*S*, not significant (*P* > 0.05). *P < 0.01. **P < 0.001. (t-test). Scale bars, 20 μm.

Knockdown of Cora using *ptc-Gal4* resulted in lethality, while *cora RNAi* by *MS1096-Gal4* can survive till adulthood. Since flies doubly knockdown by *MS1096-Gal4* (*MS1096>cora RNAi/Tctp RNAi*) can survive until the pupal stage, we examined the effects of double knockdown in third instar larval wing discs. Wing discs were stained by anti-GFP and Phalloidin (Phal) to visualize the pattern of *MS1096-Gal4* and F-actin, respectively. *MS1096*>*GFP*/+ control showed GFP staining mainly in the wing pouch area. Phal staining was relatively even in the wing pouch. Wing discs with *Tctp RNAi* or *cora RNAi* were slightly reduced in size ((90%, *n* = 13 and 85%, *n* = 14) of control size, respectively) (Fig 6B–6C”’ and 6E). Confocal sections through the medial part of Cora-depleted wing discs showed more severe Phal aggregation than those of *Tctp RNAi* (Fig 6C”, arrows).

**Fig 7:**
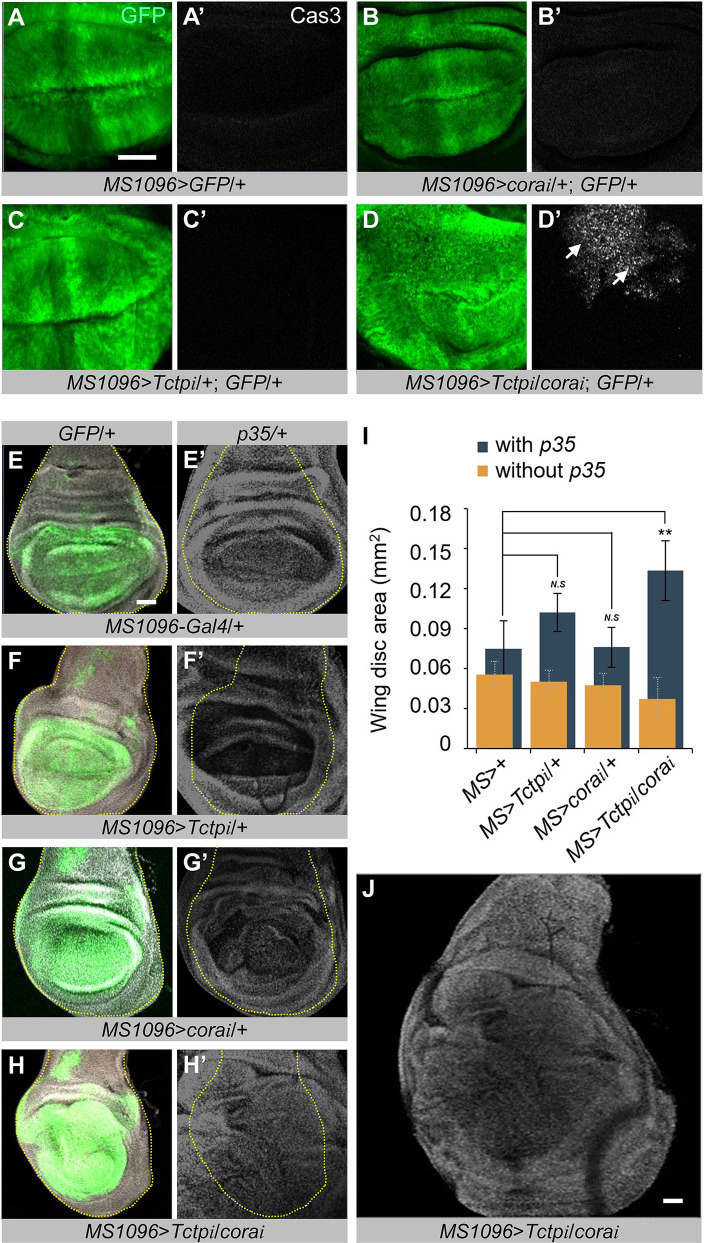
*cora-Tctp* double RNAi with cell death inhibition causes tumorous growth in the wing disc. (A-D’) Effects of *cora*/*Tctp RNAi* by *MS1096*-*Gal4* on cell death. Wing discs were stained for GFP (green) and Cas3 (white). Wild-type control of *MS1096*/+ shows little Cas3 staining (A-A’). *cora RNAi* (B-B’) or *Tctp RNAi* (C-C’) has no significant effects on Cas3 level. However, the double knockdown of Cora and Tctp results in a strong increase in Cas3 staining (D-D’). Scale bar, 20 μm. (E-H’) Effects of p35 overexpression on *cora*/*Tctp* double *RNAi* by *MS1096*-*Gal4* in the wing disc. The expression region of *MS1096*-*Gal4* marked by GFP and DAPI are shown in green and white, respectively. Control wing discs (E) are slightly increased by p35 expression (E’). *Tctp RNAi* wing discs (F) are also slightly increased by p35 (F’). *cora RNAi* wing discs (G) are weakly increased by p35 (G’). Wing discs with *cora/Tctp* double RNAi (H) are greatly overgrown by p35 (H’). (I) Quantification of p35 effects on wing disc size shown in (E-H’). Wing discs with and without p35 are shown as blue and yellow bars, respectively. (J) Image of the entire wing disc shown in (H’). It shows extensive folding and bulging of wing disc areas. Error bars are s.e.m. (*n ≥* 4). *N*.*S*, not significant. **P < 0.001. (t-test). Scale bars, 20 μm.

When both Cora and Tctp were knocked down, wing discs were slightly smaller than those depleted in either Cora or Tctp (Fig 6D–D”’ and 6E). Surprisingly, however, Cora-Tctp double knockdown caused abnormal folding of the wing pouch, implying local overgrowth in the wing disc (Fig 6D). Phal staining was greatly increased at the apical and/or basal region of folded wing disc epithelia (Fig 6D”, arrows). Such folding was found in all of Cora/Tctp double knockdown wing discs, while no such folding was observed in wing discs with a single knockdown of Cora or Tctp (Fig 6F). These results suggest that both Cora and Tctp are required to suppress abnormal overgrowth with tissue folding. Cross-section of *Tctp RNAi* wing discs showed a relatively intact pattern of Arm (S5B” Fig). Depletion of Cora led to severely defective Arm pattern and abnormal nuclear positioning (S5C’–S5C”’ Fig). Such defects may lead to abnormally uneven epithelia in the wing disc, resulting in wrinkled adult wings (S4H Fig). Wing discs with *cora/+; Tctp/+* double knockdown showed 2–3 layers of abnormally localized Arm staining (S5D’–S5D”’ Fig), which are reminiscent of abnormal Phal staining shown in Fig 6D”. Interestingly, despite the folding of wing pouch area, Cora/Tctp-depleted wing discs were relatively smaller than that of control wing discs (Fig 6E). One possibility is that double RNAi for Cora and Tctp may cause cell death in parallel with overgrowth in wing disc. To test this idea, we examined whether there is any change in the pattern of cleaved active Caspase 3 (Cas3), a cell death marker. In *MS1096*>*GFP*/+ control wing, there was no detectable Cas3 staining (Fig 7A’). Wing discs with single knockdown of Cora or Tctp also showed no significant level of Cas3 staining (Fig 7B’ and 7C’). In contrast, knockdown of both Cora and Tctp resulted in ectopic Cas3 staining within the RNAi targeted region of the wing disc (Fig 7D’). We then tested whether tissue overgrowth caused by Cora/Tctp double knockdown can be further enhanced by inhibiting cell death. Overexpression of the p35 cell death inhibitor recovered *cora* or *Tctp RNAi* wing discs to normal size or even slightly larger than normal (Fig 7F’, 7G’ and 7I), suggesting that knockdown of Cora or Tctp reduces the wing size by cell death, although cleaved Cas3 staining was not clearly detected (Fig 7B’ and 7C’). In contrast, cell death inhibition by p35 greatly increased the size of Cora/Tctp double knockdown wing discs approximately 3.5 times. The area of GFP-expressing wing pouch also showed massive overgrowth as indicated by severe bulging and tissue folding (Fig 7H’, 7I and 7J). These results support the idea that overgrowth of wing disc caused by *cora/Tctp* double RNAi is restricted by extensive cell death. Thus, inhibition of cell death strongly enhances overgrowth, leading to enlarged wing discs.

In contrast to the strong effects of loss of Tctp in development, Tctp overexpression causes no obvious phenotype in adult bodies [12]. Wing discs with Tctp overexpression by *MS1096-Gal4* were smaller than wild-type (S6A–S6B” and S6E Fig) but developed to normal wings (S6F and S6G Fig). Interestingly, Tctp overexpression in *cora RNAi* condition caused some tissue folding with increased wing disc size in 67% (*n* = 6) wing discs (S6D–S6D” and S6E Fig). However, unlike *Tctp RNAi* that causes wing disc overgrowth and pupal lethality in *cora RNAi* background, Tctp overexpression did not lead to lethality or significantly rescued *cora RNAi* adult wing phenotype, although there was mild suppression (S6H and S6I Fig).

### Depletion of both Cora and Tctp disrupts eye-head development

We also examined the relationship between Cora and Tctp localization in the eye disc. In the wild-type eye disc, Cora and Tctp showed similar patterns of immunostaining in the peripodial epithelium (S7A Fig). In eye disc proper, Tctp staining also overlapped with Cora in the membrane of undifferentiated cells anterior to the morphogenetic furrow (S7A’ Fig) and differentiating ommatidia (S7A” Fig). We then examined the effects of Tctp reduction on the Cora pattern in eye disc. Since *Tctp* null mutant clones were too small for reliably assessing the level of Cora, we used *Tctp RNAi* clones generated by the flp-out method [36]. *Tctp RNAi* clones in the eye disc did not show detectable changes in the pattern of Cora (S8A–S8A” Fig), as in embryo. Consistent with earlier studies [30], *cora* loss-of-function clones in eye discs were also very small and showed reduced levels of Tctp (S8B and S8B’ Fig).

Next, we tested whether *cora/Tctp* double RNAi by *eyeless (ey)-Gal4* in developing eye disc can induce overgrowth as in the wing. The knockdown of Tctp or Cora by *ey-Gal4* resulted in a mild reduction of the eye (S7B–S7D Fig). Remarkably, RNAi for both Cora and Tctp (*ey*>*Tctp RNAi/cora RNAi*) resulted in 100% (*n* > 43) lethality during late pupal stage. Dead pupae from *cora/Tctp* double knockdown showed nearly normal development of thorax and abdomen but lost almost all parts of the eye and head (S7E Fig). *Tctp RNAi* or *cora RNAi* caused a reduction of eye disc (S7G and S7H Fig). In striking contrast to wing overgrowth, double knockdown of Cora and Tctp resulted in small eye discs (17%, n = 12) or near complete loss of the eye disc (42%), consistent with the headless phenotype in adult flies (S7E and S7I Fig). We also tested whether p35 cell death inhibitor can suppress the defects of the eye disc. Unexpectedly, expression of p35 in *cora RNAi* eye discs resulted in loss of eye discs for unknown reason. However, p35 overexpression in *cora/Tctp* double RNAi eye discs slightly reduced the frequency of no-eye disc phenotype, suggesting a mild suppression (S9E and S9F Fig). Distinct phenotypes of *cora/Tctp* double RNAi in wing and eye suggest that Cora and Tctp functions might depend on the developmental contexts of different organs.

### Tctp interacts with other members of the Cora protein complex

**Fig 8:**
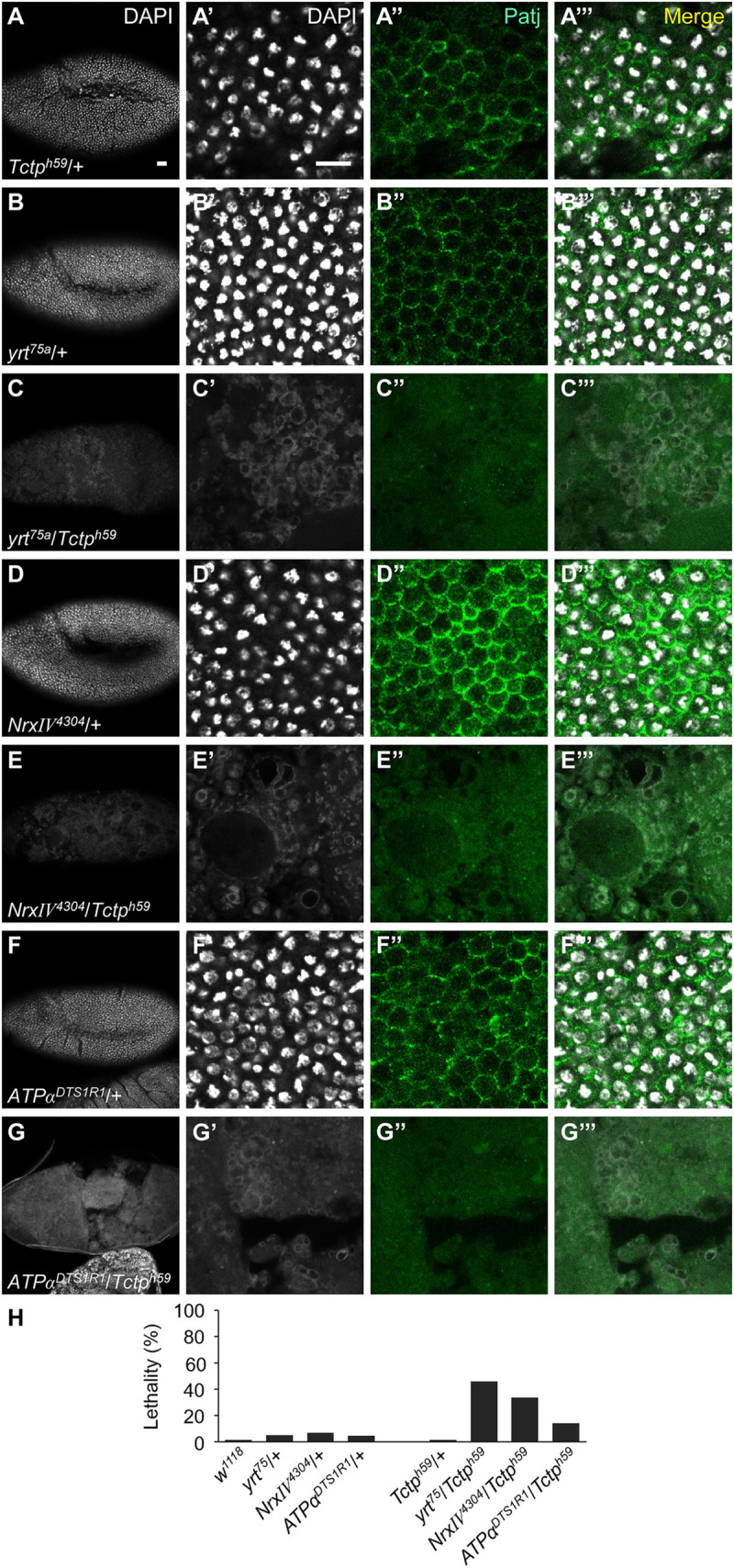
Tctp shows synergistic genetic interaction with Cora partner genes. (A-G”’) Embryos at stage 9 stained with DAPI (white) and anti-Patj antibody (green). Genotypes are as indicated. Tangential sections of embryos are shown next to the first column. Heterozygotes *Tctp*^*h59*^*/+* (A-A”’), *yrt*^*75a*^*/+* (B-B”’), *Nrx-IV*^*4304*^*/+* (D-D”’) and *ATPα*^*DTS1R1*^*/+* (F-F”’) show normal pattern of apical Patj staining. *yrt*^*75a*^ and *ATPα*^*DTS1R1*^alleles are strong hypomorphs while *Nrx-IV*^*4304*^ is amorph (FlyBase). Double heterozygotes *Tctp/yrt* (C-C”’), *Tctp/Nrx-IV* (E-E”’) and *Tctp/ATPα* (G-G”’) show severe disruption of DAPI and Patj pattern. (H) Quantification of synthetic lethality for indicated genotypes. (*n ≥* 54). Scale bars indicate 20 μm.

Cora forms a septate junction protein complex with other components, including the FERM domain protein Yrt, ATPα, and Nrx-IV. Yrt is involved in antagonizing the apical Crb complex to regulate the apicobasal epithelial polarity [37]. Hence, we tested whether Tctp genetically interacts with these Cora partners. Embryos heterozygous for *yrt*^*75*^, *Nrx-IV*^*4304*^ or *ATPα*^*DTS1R1*^ mutation were relatively normal. However, *yrt*^*75*^*/Tctp*^*h59*^ double heterozygotes showed significant embryo lethality (46%, *n* = 54) with massive disruption of epidermal and internal tissues (Fig 8C–8C”’), as shown in *cora*^*4*^*/+; Tctp*^*h59*^*/+* double heterozygotes. *Nrx-IV*^*4304*^*/Tctp*^*h59*^ and *ATPα*^*DTS1R1*^*/Tctp*^*h59*^ heterozygotes also showed similar embryonic phenotypes (Fig 8E–8E”’ and 8G–8G”’), although embryonic lethality in *ATPα*^*DTS1R1*^/*Tctp*^*h59*^ double heterozygotes occurred at a lower frequency than other Cora partner genes. These results support that *Tctp* shows similar genetic interaction with *cora* and its interacting partner genes.

**Fig 9:**
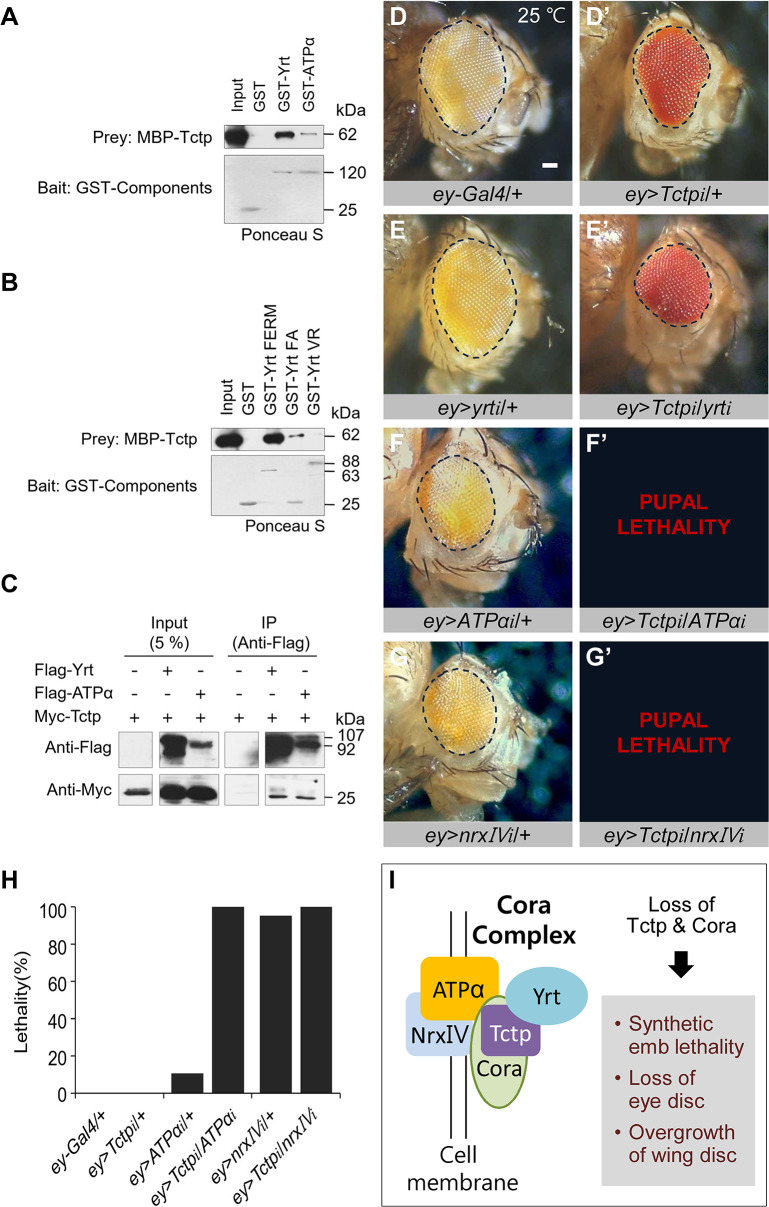
Tctp interacts with other Cora complex proteins. (A) GST-pulldown of Tctp with Yrt and ATPα. GST-Yrt and GST-ATPα bind to MBP-Tctp. (B) Tctp binds to Yrt N-terminal FERM and FERM Adjacent (FA) domain but not to C-terminal VR domain. (C) Co-immunoprecipitation of Tctp with Yrt and ATPα. Myc-Tctp is coimmunoprecipitated with Flag-Yrt and Flag-ATPα in S2 cell extracts. (D-G’) Genetic interaction of Tctp and Cora partners at 25°C. *ey*>+ control (D). *Tctp RNAi* shows reduced eye (D’). *yrt RNAi* shows normal eye (E) but enhances *Tctp RNAi* eye phenotype (E’). *ATPα RNAi* causes about 11% (*n* = 37) lethality. Most flies show small eye phenotype (F). Double RNAi for Tctp and ATPα leads to 100% (*n* = 41) lethality (F’). *Nrx-IV RNAi* by *ey*-*Gal4* causes semi-lethality. Escapers show reduced eyes (G). Double knockdown of Tctp and Nrx-IV results in 100% (*n* = 40) lethality (G’). (H) Quantification of lethality shown in (D, F, G) (*n ≥* 37). Scale bar, 100 μm. (I) A model for Tctp interaction with Cora complex proteins. Cora is required for the maintenance of Tctp. Tctp may stabilize the Cora complex by interacting with Yrt, ATPa and NrxIV. Cora and Tctp may act redundantly to regulate junctional integrity. Hence, the reduction of both Cora and Tctp results in tissue-specific defects such as embryo lethality, loss of eye disc, and overgrowth of the wing disc.

Based on their strong genetic interactions, we examined whether Tctp physically interacts with Cora partner proteins. GST-pulldown assays showed that Tctp can bind directly to Yrt and ATPα (Fig 9A). Mammalian TCTP can also bind to the Na^+^, K^+^-ATPase α subunit to inhibit the ion pump activity in cultured cells [38], although its junctional role in epithelial tissues and organs is unknown. Our data also indicate that human TCTP can bind to homologs for Cora (EPB41L3) and Yrt (EPB41L5). Interestingly, TCTP-EPB41L5 binding was stronger than TCTP-EPB41L3 (S10A Fig). We could not determine whether Tctp can also bind to Nrx- IV due to unknown difficulties in expressing intact Nrx- IV. Binding assays with truncated Yrt proteins indicated that Tctp binds to the N-terminal FERM and FERM-adjacent (FA) domains but not to the C-terminal variable region (VR) domain of Yrt (Fig 9B). Co-IP assays using S2 cells showed that Tctp co-immunoprecipitates with Yrt and ATPα (Fig 9C). In contrast, Tctp did not bind to the intracellular domain of Crumbs (Crb^intra^) and another septate junction protein Discs-large (Dlg) (S10B Fig), suggesting that Tctp binding with Cora complex proteins is selective.

We also checked whether *Tctp* and *yrt* are functionally related in organ development. Knockdown of *yrt* in eye disc using *ey-Gal4* did not cause any visible defects in the eye (Fig 9E), indicating that partial loss of Yrt alone is not critical for eye growth. However, *yrt RNAi* enhanced the eye phenotype of *Tctp RNAi* (Fig 9E’). In the wing, knockdown of Tctp or Yrt by *ptc-Gal4* showed similar reduction of the *ptc* domain between L3 and L4 veins. Double knockdown of Tctp and Yrt resulted in lethality at the pupal stage (S11A–S11D Fig), indicating strong genetic interaction between the two genes. ATPα knockdown by *ey*-*Gal4* resulted in only 11% (*n* = 37) pupal lethality. 46% of survived adult flies showed mildly reduced and rough eyes (Fig 9F and 9H) and the others were almost normal. Under this condition, *Tctp RNAi* increased pupal lethality of *ATPα RNAi* to 100% (*n* = 41) (Fig 9F’and 9H), implying strong interaction between the two genes. The knockdown of Nrx-IV by *ey*-*Gal4* resulted in late pupal lethality in more than 95% of progeny (*n* = 44) (Fig 9H). Nearly all escapers showed a reduction in the eye size (Fig 9G). Additional *Tctp RNAi* together with *Nrx-IV RNAi* caused 100% (*n* = 40) pupal lethality (Fig 9G’and 9H). Due to the high level of lethality by *Nrx-IV RNAi* alone, it was unclear whether *Tctp RNAi* synergistically enhances the effects of *Nrx-IV RNAi*. In order to reduce the lethality, we knocked down Nrx-IV at 18 ^o^C to reduce the Gal4 activity. At 18 ^o^C, about 40% (*n* = 26) of *Nrx-IV RNAi* flies died during late pupal stage while escaper adult flies (15%) showed weakly reduced and rough eyes (S11F Fig). While *Tctp RNAi* flies developed to adulthood with nearly normal eyes (S11J Fig), double knockdown of Tctp and Nrx-IV resulted in earlier pupal lethality (67%, *n* = 18). In addition, escaper double RNAi flies showed strongly reduced eyes (S11K Fig). Hence, Tctp physically and genetically interacts with Cora and its septate junction partners during eye development.

Since septate junctions are also associated with Dlg-Scrib-Lgl complex proteins, we checked whether *Tctp* shows genetic interaction with Dlg complex genes. RNAi for Dlg, Scrib or Lgl by *ey*-*Gal4* caused nearly 100% lethality (*n* ≥ 30) at 25 ^o^C. Hence, we tested genetic interaction of *Tctp* with Dlg complex genes at 18 ^o^C. Flies with *lgl RNAi* were fully viable with no obvious morphological defects in the eye. *Tctp RNAi* did not affect *lgl RNAi* flies, indicating little genetic interaction between these two genes (S11G and S11L Fig). *scrib RNAi* resulted in semi lethality (81%, *n* = 57) during late pupal or embryonic stages. Escaper adult flies showed relatively normal eyes (S11H Fig). *Tctp/scrib* double RNAi led to similar semi-lethality (89%, *n* = 54) but all died during embryonic stage. Escaper adult flies showed slightly reduced and roughened eyes (S11M Fig). Hence, *Tctp RNAi* weakly enhances the lethality and the eye phenotype of *scrib RNAi*. *dlg RNAi* caused a lower lethality (61%, *n* = 36) than *scrib RNAi* (S11I and S11O Fig). However, *Tctp* double RNAi strongly enhanced the lethality of *dlg RNAi* (89%, *n* = 40) (S11N and S11O Fig). Thus, although Tctp does not directly binds to Dlg (S10B Fig), *Tctp* shows genetic interaction with Dlg complex, preferentially through *dlg* but not *lgl*.

## Discussion

We have shown that Tctp functions together with the septate junction protein Cora to maintain epithelial integrity during embryogenesis. In contrast to the relatively normal development of *cora*^*4*^*/+* or *Tctp*^*h59*^*/+* heterozygous embryos, double heterozygous embryos undergo massive disruption in epidermal and internal structures. This suggests that Cora-Tctp interaction is sensitive to the dosage of each gene, and that their functions are severely impaired when *cora/+* and *Tctp/+* heterozygous conditions are combined, leading to synthetic embryonic lethality. Double heterozygous embryos showed a normal pattern of Dlg and Patj along the lateral plasma membrane during cellularization (Fig 3B and 3B’), suggesting that apical basal cell polarity is not significantly affected at this stage. However, double heterozygous embryos undergo rapid deterioration after cellularization (Fig 4D–4E”’ and 4G–4G”’). In particular, the regions of invagination during germ-band extension at stage 9 show various tissue damages such as bulging of germ-band and near separation of the cephalic region from the posterior body. At this time, the level of Patj is strongly reduced in the area of invagination, suggesting a defective apical-basal pattern of epithelia (Fig 4D” and 4E”). By stage 16, most of double heterozygous embryos show severe disruption of epidermal and interior structures of the embryo, resulting in lethality prior to cuticle formation (Fig 2I and 2J). We noted in double heterozygous embryos that Dlg staining is considerably enhanced with broader distribution while Patj staining is decreased (S2D–S2D”’ Fig). These data suggest that other septate junction components like Dlg might be affected by the simultaneous reduction of Cora and Tctp. Analysis of mutant embryos showed that epidermal localization of Tctp is reduced in *cora*^*4*^ mutant embryos. On the contrary, *Tctp* mutant embryos show nearly normal localization of Cora. Hence, maintenance of Tctp levels depend on Cora but not *vice versa*, although we do not exclude the possibility that Cora localization might also be affected by Tctp in a tissue-specific manner.

Our data also show synergistic genetic interactions between *cora* and *Tctp* in organ development after embryogenesis. Cora and Tctp together are essential for eye-head development (S7B–S7I Fig), consistent with their roles in cell proliferation. On the contrary, our work provides evidence that simultaneous reduction of Cora and Tctp can induce synthetic overgrowth in the wing (Figs 6D–6F and 7H–7J), suggesting that Cora and Tctp can function together as tumor suppressors. Arm and Phal staining of double RNAi wing discs suggest abnormalities in adherens junctions and the actin cytoskeleton. It is yet to be determined whether defective epithelial integrity is causally related to the overgrowth of wing disc. Several possibilities can be considered for the role of Cora and Tctp in the regulation of wing growth. Firstly, Cora and Tctp might indirectly regulate the tumor suppressor function of the Dlg complex. It has been shown that apical aPKC is mutually exclusive to the basolateral localization of the Yrt complex [39]. Hence, the reduction of Cora and Tctp might lead to an increased activity of the apical proteins, thus indirectly reducing the tumor suppressor function of the Dlg complex. Secondly, Tctp might interact with both the Cora and Dlg complexes. In this case, knockdown of Cora and Tctp might disrupt the Cora complex, impairing epithelial integrity. In parallel, it may also affect the tumor suppressor function of the Dlg complex in wing disc, resulting in tissue overgrowth. Both of these possibilities seem to be consistent with our data for genetic interaction between *Tctp* and *dlg*. Thirdly, overgrowth in *cora/Tctp* double RNAi wing discs may be induced by dying cells to compensate the loss of cells in developing wing discs. Dying cells in imaginal discs can induce non-cell autonomous signaling for proliferation in neighboring cells [40, 41, 42, 43]. Hence, overexpression of p35 in *cora/Tctp* double RNAi wing discs may generate undead cells, inducing extensive overgrowth (Fig 7H’–7J). If *cora*/*Tctp* double RNAi induces compensatory proliferation, we would expect that overgrowth is likely to occur mainly in GFP-negative cells adjacent to the GFP-positive Gal4-driven RNAi target cells. However, our data show that *cora*/*Tctp* double RNAi induces the most prominent overgrowth in GFP-positive cells (Fig 6D–6D”’). Hence, overgrowth by Cora/Tctp double knockdown appears to be different from compensatory proliferation, although we cannot exclude the possibility.

An intriguing feature of Cora and Tctp functions is their synergistic genetic interaction. Synthetic lethality of double heterozygous embryos and severe imaginal disc defects by double knockdown raise possibility that Cora and Tctp may have partially redundant functions. Such synergistic genetic interaction might also occur by differential effects of partial loss of individual component in a protein complex. Since Cora forms a complex with Yrt, Nrx-IV, and ATPα, partial loss of Cora by *cora/+* heterozygous mutations or RNAi may destabilize the Cora complex, thus sensitizing the complex without disrupting its function. Our data show that Tctp not only binds to Cora but also Yrt and ATPα (Fig 9A). Thus, an addition of *Tctp* mutation in such sensitized *cora/+* genetic background may disrupt the Cora complex function, resulting in severe synergistic defects in adult organ development (S4I and S7E Figs). Similar synergistic genetic interaction of *Tctp* with *yrt*, *Nrx-IV*, and *ATPα* was also found in embryo development (Fig 8). Hence, Tctp seems to affect Cora complex function through physical interaction with multiple Cora partners in different tissues. It is an interesting question how *cora*/*Tctp* double heterozygous mutations or double knockdown lead to loss of tissues in eye disc while resulting in overgrowth in the wing disc. Cancer can occur by activation of oncogenes or inactivation of tumor suppressor genes. However, recent studies suggest that there are hub genes or double agents that can function as proto-oncogenic and tumor suppressor depending on tissue contexts. Such double agents play a major role in inducing cancer [44]. For instance, mammalian *Notch* acts as both oncogene and tumor suppressor in different cellular environments [45, 46]. Tumor suppressors like *fat* and *lgl* are required for normal oogenesis, although they are required for restricting cell proliferation [47]. Since subcellular distribution of Tctp varies in different organs, the effects of Cora-Tctp interaction may depend on different interacting partners in different types of tissues and organs.

While Cora is the only 4.1 family protein in *Drosophila*, at least four Protein 4.1 family genes (*EPB41* and *EPB41L1-L3*) have been identified in vertebrates. Among these, 4.1B and 4.1R proteins are involved in tumor suppression and metastasis in diverse types of cancer [48]. Our data show that human TCTP also physically interacts with 4.1 family proteins such as EPB41L3 (Cora ortholog) and EPB41L5 (Yrt ortholog). It is an intriguing question whether mammalian Tctp interacts with these 4.1 family proteins to synergistically regulate epithelial integrity and tissue growth. The proposed new roles of *Drosophila* Tctp in the regulation of epithelial organization and growth may provide insights into mammalian TCTP functions in development and growth-related diseases.

## Materials and methods

### *Drosophila* genetics

All flies were raised at room temperature (RT). Genetic crosses were made at 25°C unless stated otherwise. The wild-type control was *w*^*1118*^ strain. *cora RNAi* (v9787), *yrt RNAi* (v28674), and *ATP*α* RNAi* (v12330) were obtained from the Vienna Drosophila Resource Center (VDRC). *Nrx-IV RNAi* (18353), *cora*^*2*^ (58805), *Df(2R)Exel6069 (56B5-56C11)* (7551), *Nrx-IV*^*4304*^ (4380) were from the Bloomington Drosophila Stock Center (BDSC). *Tctp RNAi* and *Tctp*^*h59*^ *FRT82B/TM6 Tb* mutant were as described [12]. *cora*^*4*^ strong hypomorph mutant strain (*yw*; *b pr cora*^*4*^ *FRT 43D*/*CyO*) was from Dr. Rich Fehon (University of Chicago, USA). *yrt*^*75*^ was from Dr. Ulrich Tepass (University of Toronto, Canada), and *ATPα*^*DTS1R1*^ was from Dr. Michael Palladino (University of Pittsburgh School of Medicine, USA). Mitotic clones were generated in imaginal discs by using *ey-flp*.

To generate *cora/+; Tctp/+* double heterozygous embryos, we used 2^nd^ and 3^rd^ chromosome balancer stocks. UAS-2xEGFP driven by a *twist*-*Gal4* driver from Dr. Seyeon Chung (Louisiana State University, USA) as a selection marker. Balancer chromosomes are described at FlyBase [49].

For double knockdown of Cora and Tctp, *ey*>*Tctp RNAi*/*CyO*, *GFP* and *ptc*>*Tctp RNAi*/*CyO*, *GFP* recombinants were crossed with *UAS-cora RNAi*. Double knockdown of *Tctp* and other genes were similarly performed. To check the effects of double knockdown in the tissues expressing *MS1096-Gal4*, we first generated *MS1096*/+; *cora RNAi*/*In(2LR)Gla, wg[Gla-1] Bc[1]*; *UAS-GFP*/+ and selected GFP expressing larvae. The progeny with GFP expression and without *BC* balancer was crossed with flies with *Tctp RNAi* inserted on the second chromosome.

### Cuticle preparation

Cuticles from embryos and first instar larvae were prepared using the protocol described in [50] with minor modification. Eggs were collected for 4 h on grape juice plates with yeast paste and incubated for 24 h at 25°C. Late-stage embryos and 1^st^ instar larvae collected were washed on the mesh with distilled water. The 1:1 mixture of lactic acid and Hoyer’s mount solution was added as soon as the water was removed. After 1 min, the samples on the slide glass were mounted with a cover glass and incubated overnight at 65°C.

### Plasmid construction and *In vitro* GST-pull down assay

Tctp was tagged with MBP using pMAL-c2 vector [19]. cDNAs for Cora (DGRC, IP14940; isoform PC), Yrt (DGRC, LD33734; isoform PA), ATPase α (DGRC, GH23483), Yrt fragments (Yrt FERM, Yrt FA, Yrt VR), Crb^intra^ [51], Dlg (DGRC, RE30311; isoform PG) were cloned into bacterial expression vector (pGEX4T-1 vector) using In-Fusion HD cloning kit (Clontech). Cora isoform PC consists of 703 amino acids including the N-terminal FERM domain and a C-terminal domain, and it is sufficient to rescue *cora* mutations [32, 35]. Yrt deletion constructs were generated by PCR using In-Fusion HD Mutagenesis Kit (Clontech). Yrt fragments for FERM region (703–1727 AAs, 37.6 kDa), FA region (1728–1900 AAs, 6.3 kDa) or variable region (1901–3622 AAs, 63.1 kDa) were amplified for 25 cycles of 3 min at 95°C and 10 s at 98°C, followed by 10 s at 55°C and 10 min at 68°C. Amplified DNA constructs were confirmed by DNA sequencing. Human EPB41L3 (Cora homolog, Genomics-online, ABIN4830867), human EPB41L5 (Yrt homolog, Genomics-online, ABIN4830869) were cloned into His-GST vector.

All proteins were expressed in *E*. *coli* Rosetta2 cells by induction with 0.1 M IPTG using the manufacturer’s protocol (NEB and GE healthcare, USA) at 15°C overnight. Diluted cells were incubated in Terrific Broth [52]. Cell pellets were resuspended in ice-cold PBS containing proteinase inhibitor and phosphatase inhibitor cocktail (Roche). 10% Triton X-100 was added in sonicated cells (final 1% in PBS) and incubated with sepharose 4B resin. The resin was washed in PBS containing 1 mM EDTA and 1 mM PMSF. GST-fusion proteins were eluted in Tris/GSH elution buffer (50 mM Tris-HCl (pH 8.0), 150 mM NaCl, 5 mM DTT, 10 mM reduced GSH). Purified proteins were dialyzed in dialysis buffer (20 mM Tris (pH 8.0), 20% glycerol, 150 mM NaCl and 1 mM DTT) at 4°C overnight. Proteins were concentrated using Amicon Ultra Centrifugal Filters (Merck Millipore). MBP-tagged Tctp was used as prey and GST-tagged proteins as baits for GST-pull down. The same amount (10 μg/mL) of prey and bait proteins were incubated in PDB buffer (20 mM Tris-HCl (pH 7.5), 150 mM NaCl, 0.5 mM EDTA, 10% glycerol, 0.1% Triton X-100, 1 mM DTT, protease inhibitor cocktail (Roche), and 1 mM PMSF).

### Immunohistochemistry

#### Embryo staining

To check early stages of embryogenesis including the cellularization steps, embryos were collected on grape juice plates for 4 h and immunostained. For later stages, embryos were collected for 4 h, incubated for additional 14 h, and immunostained. Embryo fixation was done by a standard method known as Slow Formaldehyde Fixation [53, 54]. Embryos were collected using 1 X TXN buffer and dechorionated by 50% bleach. 2 mL of heptane was used to wash the embryos off the mesh into the vial. An equal volume of 3.7% formaldehyde in PEM buffer (PIPES-EGTA-Magnesium chloride) was added immediately. Embryos were incubated for 20 min at RT. PEM buffer was replaced with methanol for devitellinization. After removing all solution including the upper heptane layer, embryos were rinsed by methanol three times and stored in ethanol at -20°C.

For embryo staining, embryos were incubated in 0.2% saponin/PBS in a rotator 10 min twice at RT. Primary antibodies were diluted in 0.2% saponin/0.5% goat serum/PBS and incubated overnight at 4°C. After washing for 15 min four times at RT, embryos were incubated in secondary antibodies overnight at 4°C. Antibodies were used as follows: sheep anti-GFP (Ab-direct Serotec 4745–1051) at 1:200, rabbit anti-Tctp at 1:200, mouse anti-Cora (1:100; DSHB, C566.9), mouse anti-Arm (1:100; DSHB), rabbit anti-Dlg (1:500; gift from Dr. Kyung-Ok Cho, Korea Advanced Institute of Science and Technology, Korea), mouse anti-Patj (1:200; gift from Dr. Hugo Bellen, Baylor College of Medicine, Houston, TX). Secondary antibodies conjugated with FITC, Cy3, and Cy5 (Alexa Flour, Jackson Immunoresearch) were used. Anti-sheep FITC and anti-mouse Cy3 were diluted at 1:200. Anti-rabbit Cy5 was used at 1:500. Stages of stained embryos were determined based on the morphological features described in the FlyBase [49].

#### Imaginal discs

Immunostaining of imaginal discs was according to a modified protocol from Carroll & Whyte’s [55]. Third instar eye and wing imaginal discs were fixed with PLP (2% paraformaldehyde) for 15 min at RT. After PBS washing twice, the discs were blocked in solution containing 0.02% NaN_3._ Primary antibodies were incubated overnight at 4°C. Samples were washed for 40 min four times at RT. Secondary antibodies were incubated for 2 h at RT. The following primary antibodies were used: guinea pig anti-Cora (1:10000; gift from Dr. Rich Fehon, University of Chicago, USA), rabbit anti-Tctp (1:200), sheep anti-GFP (1:200), rabbit anti-Cas3 (1:200), mouse anti-Arm (1:100), rabbit anti-Phospho histone 3 (1:100). Secondary antibodies were: anti-mouse FITC (1:200), anti-rabbit Cy3 (1:200), anti-sheep FITC (1:200), anti-guinea pig Cy5 (1:200). Phalloidin was used at 1:200. After four times washing for 60 min, discs were incubated in DAPI solution for 10 min and washed in PBS twice for 5 min each. The samples were mounted with Vectashield (Vector) solution and stored at -20°C.

### Immunoprecipitation

For transient transfection, S2 cells were grown in M3 media (Sigma) with 10% insect medium supplement (Sigma) or M3 media with FBS (GE, SH30084.03). DNA constructs for transfection were prepared by PCR amplification from pMal-Tctp plasmid and other cDNA clones obtained from the Drosophila Genomics Research Center (DGRC). The insert fragments were cloned into pAc5.1 vector derivatives (Invitrogen) using the In-Fusion cloning kit. Transfection was carried out with Effectene reagent (Qiagen) and DNA constructs adjusted to 1 μg/μL. S2 cells collected after 24 h of transfection and embryo extracts homogenized were washed in PBS and lysed in cold lysis buffer (20 mM HEPES (pH 7.5), 100 mM KCl, 2.5 mM EDTA, 5% glycerol, 1 mM DTT, 0.05% Triton X-100, proteinase inhibitor, and phosphatase inhibitor cocktail) for 30 min at 4°C. After preclearing with protein G-sepharose beads (Roche), binding complexes were immunoprecipitated with rabbit anti-c-MYC (Abcam, Ab9106) or rabbit anti-Tctp-protein G agarose beads at 4°C overnight. The immunoprecipitates were washed three times with cold IP buffer at 4°C. The samples were boiled in protein loading buffer at 94°C for 5 min and run on gel electrophoresis for western blotting.

### Immunoblotting

Embryo protein extracts were prepared by lysing dechorionated embryos using homogenizer in SDS sample buffer, and boiled at 94°C for 5 min. Proteins from S2 cells and *E*.*coli* R2 cells were separated by gel electrophoresis and transferred onto nitrocellulose membrane. Proteins from embryos were transferred onto PVDF membrane. Membrane blots from S2 or R2 cells were blocked in 5% skim milk (Biorad). Embryo protein blots were blocked in 3% BSA (Bovine Serum Albumin, Amresco). Blots were incubated with primary antibody overnight at 4°C. Primary antibodies were as follows: mouse anti-Flag (Sigma, F1804, 1:5,000), mouse anti-Myc (Santa cruz, 9E11:sc-47694, 1:5,000), mouse anti-Cora (DSHB, C566.9, 1:100), mouse anti-α tubulin (Sigma, T9026, 1:1,000) or rabbit anti-Tctp (1:200). After washing in TBST solution three times for 5 min each at RT, blots were incubated with anti-mouse (Jackson, 715-035-151, 1:10,000) or anti-rabbit (Jackson, 711-005-152, 1:10,000) HRP-conjugated secondary antibody for 1 h at RT. Secondary antibodies were washed with TBST three times for 10 min each at RT. Immunostaining was developed using the SuperSignal West Pico Chemiluminescent Substrate kit (Thermo Scientific).

### Imaging and Statistical analysis

All fluorescent images were acquired using Carl Zeiss LSM 710 or LSM 780 confocal microscope and ZEN software. Each fluorescence laser intensity was adjusted based on the imaging of a control sample, and experimental samples were scanned using the same laser intensity. To compare tangential images of embryos, we set the top (dorsal) and bottom (ventral) of each sample and sectioned tangentially with the same thickness. For comparing cross-section views, section images obtained as described above were vertically sectioned using the orthogonal imaging tool of the Zen program. Cross-sections were made along the anterior-posterior axis of embryo. Wing discs were sectioned tangentially from top (apical) to bottom (basal) of the epithelia. For cross-section views, collected tangential images were vertically sectioned along the anterior-posterior axis of the wing pouch. Cross-sections were made at several different dorso-ventral positions. Control and experimental samples were compared using the sections acquired from the same regions of embryos and discs.

Embryo cuticle samples, adult eyes, and wings were serially photographed from top to bottom with an Axioscope camera with Axiocam software (Zeiss). Multi-level images were combined using Zeren Stacker software. The size of wing discs was quantified using Image J software. Western blot images were taken as photographs saved in JPEG files. Each lane was quantified and normalized to the loading control using the Image J program. Statistical significance was evaluated by unpaired one-tailed Student’s t-test using Microsoft Office Excel. P-values of < 0.05 were considered as statistically significant. All data represent the mean ± s.e.m. (standard error of the means).

## Supporting information

S1 Fig Full-size western blots and effects of *cora* or *Tctp* mutation in the embryo. (A) Full-size western blots of the endogenous IP result shown in Fig 1C. Immunoprecipitated endogenous Cora and Tctp are detected at about 200 and 25 kDa, respectively. After gel transfer, the blot was divided into two for separate staining with anti-Cora and anti-Tctp antibody, respectively. Protein bands in the boxes are shown in Fig 1C. (B) Effects of *cora* or *Tctp RNAi* shown in Fig 1D. The same set of samples was run on two gels. After transfer, one blot was divided into two for staining with anti-Cora and anti-Tctp, respectively. Another blot was stained with anti-α-Tub. Endogenous Cora and Tctp are detected as about 200 and 25 kDa proteins. Bands marked by an asterisk are non-specific proteins cross-reacting with anti-Tctp and show no change by *Tctp RNAi*. Protein bands in the boxed area are shown in Fig 1D. Note that *Tctp RNAi* does not affect the level of Cora, but *cora RNAi* reduces the level of Tctp. (C-D”’) Effects of *cora*^*4*^ or *Tctp*^*h59*^ mutation in embryos. Embryos in C-C”’ and D-D”’ are the same embryos shown in Fig 1G–1G”’ and 1H–1H”’, respectively. Note that images in C-D”’ are taken at a different level with low magnification to show the midgut (yellow arrows). The boxed areas are shown in Fig 1G–1H”’. Based on the pattern of the epidermal segment and the shape of the midgut, these embryos seem to be at stage 14. Tctp levels are significantly reduced in *cora*^*4*^ mutant embryo that shows a low level of Cora (D”’). Scale bar, 50 μm.
(TIF)

S2 Fig *cora/Tctp* double heterozygous embryos show enhanced Dlg. (A-D”’) Cross-section views of stage 16 embryo epidermis stained for DAPI, Dlg and Patj. Genotypes are as indicated in the DAPI panels. Wild-type *w*^*1118*^ (A-A”’). *Tctp*^*h59*^*/+* shows enhanced Dlg staining but reduced Patj levels (B-B”’). *cora*^*4*^*/+* shows stronger Dlg staining whereas Patj staining is normally localized. (C-C”’). Double heterozygotes show stronger and broader Dlg staining while Patj staining is significantly reduced (D-D”’). Scale bar, 20 μm.
(TIF)

S3 Fig *Tctp* mutation synergistically interacts with *cora*^*2*^ and deficiency. (A-F”’) Embryos of indicated genotypes were stained for DAPI and Patj. Wild-type control (A-A”’). *Tctp*^*h59*^*/+* (B-B”’) and *cora*^*2*^*/+* (C-C”’) embryos show normal Patj staining. *cora*^*2*^*/+; Tctp*^*h59*^*/+* show severe disruption of DAPI and Patj pattern (D-D”’). *cora*^*Df*^*/+* shows normal Patj staining (E-E”’). *cora*^*Df*^*/+; Tctp*^*h59*^*/+* shows severe disruption of DAPI and Patj pattern (F-F”’). (G) Quantification of embryo lethality for indicated genotypes. Scale bars, 20 μm.
(TIF)

S4 Fig Synergistic genetic interaction between *cora* and *Tctp* in wing development. (A-A’) Immunostaining of Cora (red) and Tctp (green) in the wing disc. DAPI is shown in white. An enlarged image of a hinge region of the wing disc. Cora and Tctp overlap together in the cell membrane region. (A’) Magnification of a wing pouch region. Tctp is detected in the cytoplasm but is enriched at the membranes in a similar pattern as Cora (arrows). (B-F) Effects of RNAi driven by *ptc*-*Gal4*. Control wing with one copy of *ptc*-*Gal4* is normal (B). *cora*^*4*^ /+ heterozygous wings are slightly larger than normal (C). *Tctp RNAi* reduces wing tissue in the *ptc* domain (D). *Tctp RNAi* phenotype is enhanced by *cora*^*4*^ /+ (E). Quantification of entire wing sizes and areas of *ptc* expression region. Blue bars indicate the entire wing area, and yellow bars shows the *ptc* region between L3-L4 veins (F). Error bars are s.e.m. (*n* = 6). *N*.*S*, not significant (*P* > 0.05). *P < 0.05. **P < 0.01. ***P < 0.001. ****P < 0.0001. (t-test). (G-I) Effects of RNAi driven by *MS1096*-*Gal4*. The control wing of *MS1096*-*Gal4* is normal similar to the control wing in (B). *Tctp RNAi* results in the reduced and wrinkled wing (G). *cora RNAi* shows a very small and severely disrupted wing (H). Double knockdown of Cora and Tctp causes pupal lethality (I). White and black scale bars are 10 and 50 μm, respectively.
(TIF)

S5 Fig *cora/Tctp* double knockdown disrupts the pattern of Arm in the wing disc. (A-D”’) Cross-section views of wing discs stained with DAPI and anti-Arm antibody. Cross-sections were made along the dorsoventral boundary of the wing disc shown as a straight line in Fig 6A. Genotypes are as indicated. RNAi was induced by *MS1096-Gal4* (*MS* in short). Wild-type control shows staining at adherens junctions (A-A”’). *Tctp RNAi* shows relatively normal Arm pattern (B-B”’). *cora RNAi* causes a highly irregular Arm pattern and abnormal nuclei positions (C-C”’). *cora/Tctp* double RNAi causes abnormal positioning of nuclei and mislocalization of Arm stain to basal positions (D-D”’). Scale bar, 20 μm.
(TIF)

S6 Fig Tctp overexpression weakly suppresses *cora RNAi* wing phenotype. (A-D”) Wing discs of indicated genotypes were stained for GFP and Arm. RNAi was induced by *MS1096-Gal4*. Wild-type control (A-A”). Wing discs with Tctp overexpression are reduced in size but show normal morphology (B-B”). *cora RNAi* wing discs are reduced with abnormal morphology (C-C”). *cora RNAi* wing discs with Tctp overexpression show significant folding (D-D”). (E) Quantification of wing disc size for indicated genotypes. Error bars are s.e.m. (*n ≥* 6). *N*.*S*, not significant (*P* > 0.05). *P < 0.05. ****P < 0.0001. (t-test). (F-I) Adult wing phenotypes in control (F), Tctp overexpression (G), *cora RNAi* (H) and *cora RNAi* with Tctp overexpression (I). (*n* ≥ 11). Scale bars, 50 μm.
(TIF)

S7 Fig Cora and Tctp are synergistically required for eye-head development. (A-A”) Wild-type eye disc stained for Cora (red) and Tctp (green). Cora and Tctp stains overlap in the peripodial membranes and eye disc proper (A’). Both Cora and Tctp are enriched in interommatidial cells (arrows) and at the center of each photoreceptor clusters where photoreceptor precursors form cell junctions. Cell nuclei are marked by DAPI (white) (A”). The position of morphogenetic furrow (MF) is indicated by white arrows. Scale bars, 10 μm. (B-E) Adult eye phenotypes. Genotypes are as indicated in each panel. *ey*-*Gal4* control (B). The knockdown of Tctp shows a mild reduction of eye size (C). *cora RNAi* shows a reduced eye (D). Double knockdown of Cora and Tctp causes pupal lethality. The removal of pupal case shows loss of the eye and head structure (E). Scale bar indicates 100 μm. (F-I) 3^rd^ instar larval eye discs stained with DAPI. *ey*>+ control (F). *Tctp RNAi* reduces the size of the eye disc (G). *cora RNAi* also shows small eye disc with more severity than *Tctp RNAi* phenotype (H). Double knockdown of Cora and Tctp shows the loss of the entire eye disc (I). Scale bar, 10 μm.
(TIF)

S8 Fig Effects of *cora* mutation on Tctp level. (A-A”) *Tctp RNAi* flp-out clones in the eye disc. RNAi clones are marked by GFP-positive cells. Tctp (red) is reduced in *Tctp RNAi* clones (GFP-positive), where Cora (white) shows no obvious change. (B-B’) *cora*^*4*^ mutant clones marked by GFP-negative cells in eye disc show reduced levels of Tctp (red). Scale bars, 20 μm.
(TIF)

S9 Fig Effects of p35 overexpression in *cora/Tctp* double RNAi eye discs. (A-D”) Eye-antenna discs were stained for Arm and PH3. Genotypes are as indicated in each panel. Eye discs in the absence of p35 are shown in S7F–S7I Fig. p35 overexpression does not affect control eye disc (A-A”). p35 overexpression weakly increases *ey>Tctp RNAi* eye disc (B-B”). p35 strongly reduces *cora RNAi* eye disc without affecting the antenna disc (C-C”). p35 overexpression causes minor recovery of eye disc from no eye-disc phenotype of *cora/Tctp* double RNAi (D-D”). (E) Quantification of eye disc size with and without p35 overexpression. Error bars are s.e.m. (*n ≥* 6). *N*.*S*, not significant (*P* > 0.05). *P < 0.05. ****P < 0.0001. (t-test). (F) The percentage of eye discs showing phenotypes: none (no eye disc), severe loss (less than 25% of control eye disc size), mild loss (less than 70% of control eye disc size). (*n ≥* 6). Scale bar, 50 μm.
(TIF)

S10 Fig Tctp binding with Cora, Dlg, Crb, and human homologs. (A) GST-pulldown indicates that human TCTP binds weakly to EPB41L3 (Cora homolog) and more strongly to Yrt homolog EPB41L5. (B) GST-pulldown shows that Tctp binds to Cora but not to Crb^intra^ and Dlg.
(TIF)

S11 Fig Genetic interaction between Tctp and other septate junction proteins. (A) *ptc*>+ control. (B) *ptc*>*yrt RNAi* (*yrti*)*/+*. The region between L3 and L4 vein is reduced. (C) *ptc*>*Tctp RNAi (Tctpi)/+*. The region between L3 and L4 vein is reduced. (D) *ptc*>*Tctpi/yrti* shows pupal lethality. Scale bar, 20 μm. (E-N) Genetic interaction of Tctp and other septate junction components at 18 ^o^C. All flies are female. *ey*>+ control (E). *Tctp RNAi* causes a mild reduction in the eye (J). *ey*>*Nrx-IV RNAi* results in semi-lethality. Escapers show reduced rough eyes (F). Double knockdown of Tctp and Nrx-IV results in 94% lethality with enhanced eye phenotype (K). *lgl RNAi* shows normal eye (G) and has no effect on *Tctp RNAi* (L). *scrib RNAi* shows semi-lethality. Escapers show normal eye (H) but slightly enhances *Tctp RNAi* eye phenotype (M). *dlg RNAi* also causes semi-lethality, but the escapers show abnormal eye growth (I). Double RNAi for Tctp and Dlg leads to female lethality (N). (O) Quantification of relative viability for genotypes shown in E, G-J, L-N. The same number of males and females was used for crosses, and viability was relative to *ey>+* control. Scale bar, 100 μm. (*n ≥* 18).
(TIF)
