# Supplementary figures and images for: Regulation of epithelial integrity and organ growth by Tctp and Coracle in Drosophila
Source: PLoS Genet. 2020 Jun 19;16(6):e1008885. doi: 10.1371/journal.pgen.1008885 (PMC7329144; doi:10.1371/journal.pgen.1008885)

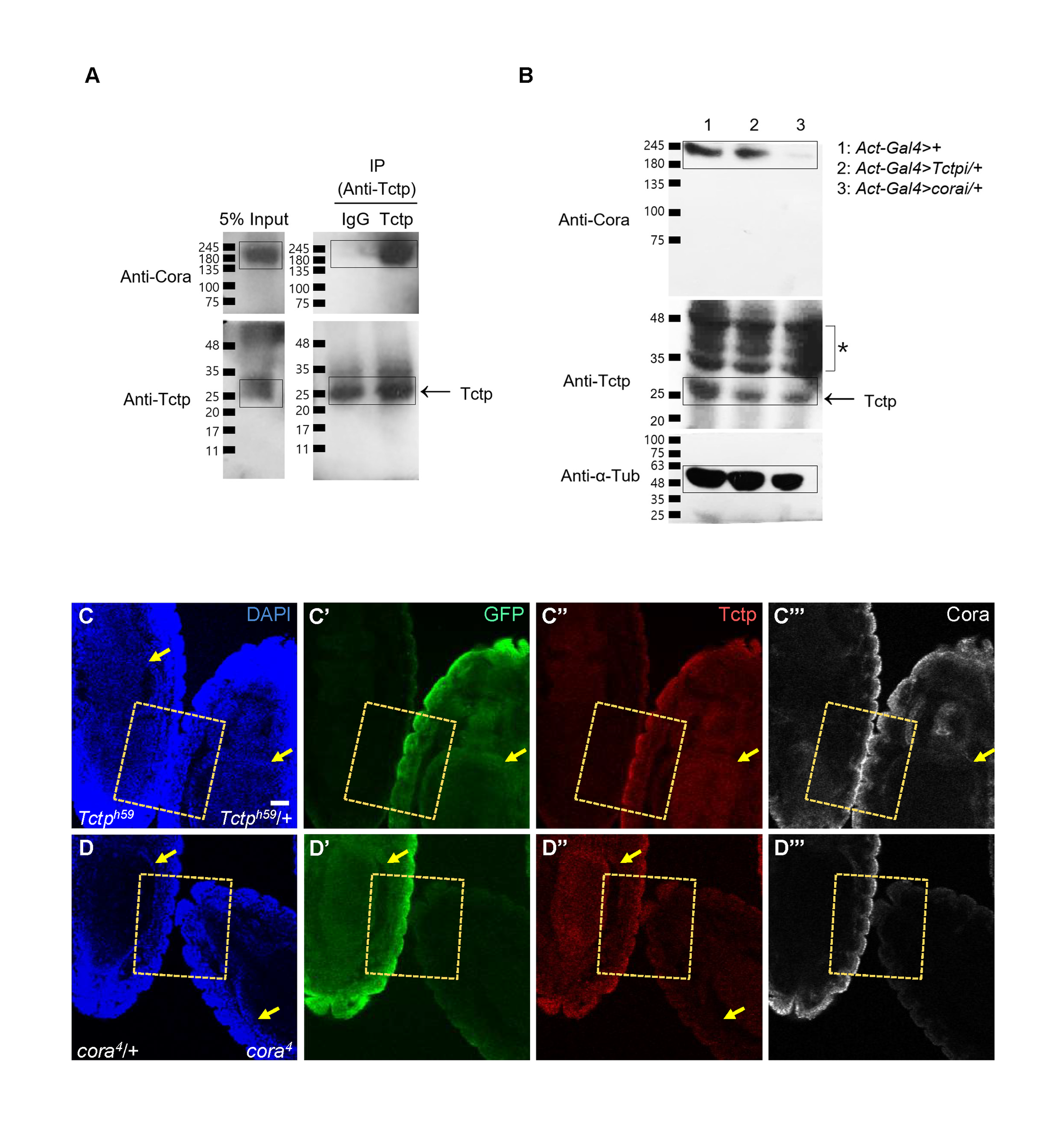

Supplement: S1 Fig — (A) Full-size western blots of the endogenous IP result shown in Fig 1C. Immunoprecipitated endogenous Cora and Tctp are detected at about 200 and 25 kDa, respectively. After gel transfer, the blot was divided into two for separate staining with anti-Cora and anti-Tctp antibody, respectively. Protein bands in the boxes are shown in Fig 1C. (B) Effects of cora or Tctp RNAi shown in Fig 1D. The same set of samples was run on two gels. After transfer, one blot was divided into two for staining with anti-Cora and anti-Tctp, respectively. Another blot was stained with anti-α-Tub. Endogenous Cora and Tctp are detected as about 200 and 25 kDa proteins. Bands marked by an asterisk are non-specific proteins cross-reacting with anti-Tctp and show no change by Tctp RNAi. Protein bands in the boxed area are shown in Fig 1D. Note that Tctp RNAi does not affect the level of Cora, but cora RNAi reduces the level of Tctp. (C-D”’) Effects of cora4 or Tctph59 mutation in embryos. Embryos in C-C”’ and D-D”’ are the same embryos shown in Fig 1G–1G”’ and 1H–1H”’, respectively. Note that images in C-D”’ are taken at a different level with low magnification to show the midgut (yellow arrows). The boxed areas are shown in Fig 1G–1H”’. Based on the pattern of the epidermal segment and the shape of the midgut, these embryos seem to be at stage 14. Tctp levels are significantly reduced in cora4 mutant embryo that shows a low level of Cora (D”’). Scale bar, 50 μm. (TIF) [file pgen.1008885.s001.tif]

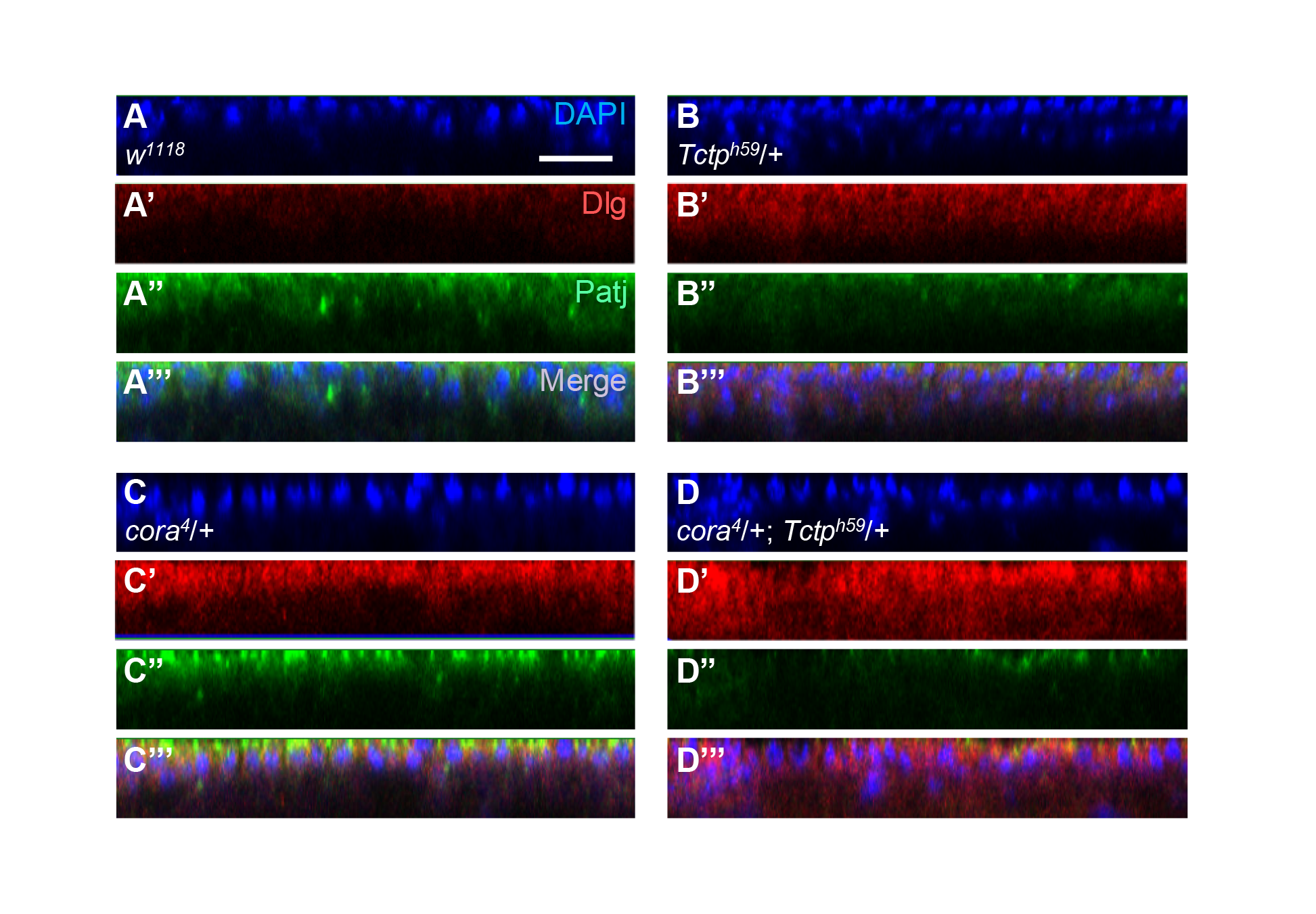

Supplement: S2 Fig — (A-D”’) Cross-section views of stage 16 embryo epidermis stained for DAPI, Dlg and Patj. Genotypes are as indicated in the DAPI panels. Wild-type w1118 (A-A”’). Tctph59/+ shows enhanced Dlg staining but reduced Patj levels (B-B”’). cora4/+ shows stronger Dlg staining whereas Patj staining is normally localized. (C-C”’). Double heterozygotes show stronger and broader Dlg staining while Patj staining is significantly reduced (D-D”’). Scale bar, 20 μm. (TIF) [file pgen.1008885.s002.tif]

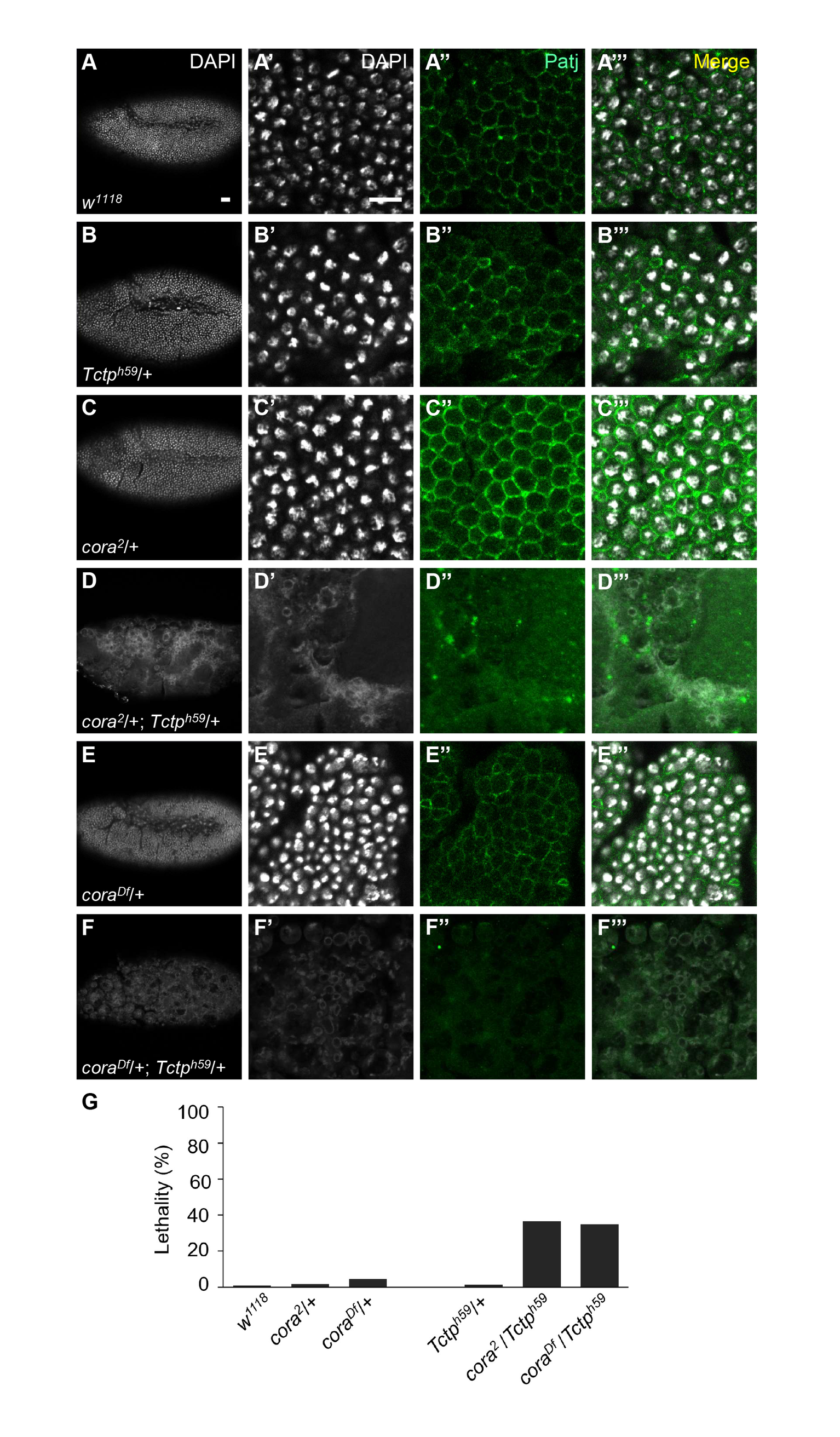

Supplement: S3 Fig — (A-F”’) Embryos of indicated genotypes were stained for DAPI and Patj. Wild-type control (A-A”’). Tctph59/+ (B-B”’) and cora2/+ (C-C”’) embryos show normal Patj staining. cora2/+; Tctph59/+ show severe disruption of DAPI and Patj pattern (D-D”’). coraDf/+ shows normal Patj staining (E-E”’). coraDf/+; Tctph59/+ shows severe disruption of DAPI and Patj pattern (F-F”’). (G) Quantification of embryo lethality for indicated genotypes. Scale bars, 20 μm. (TIF) [file pgen.1008885.s003.tif]

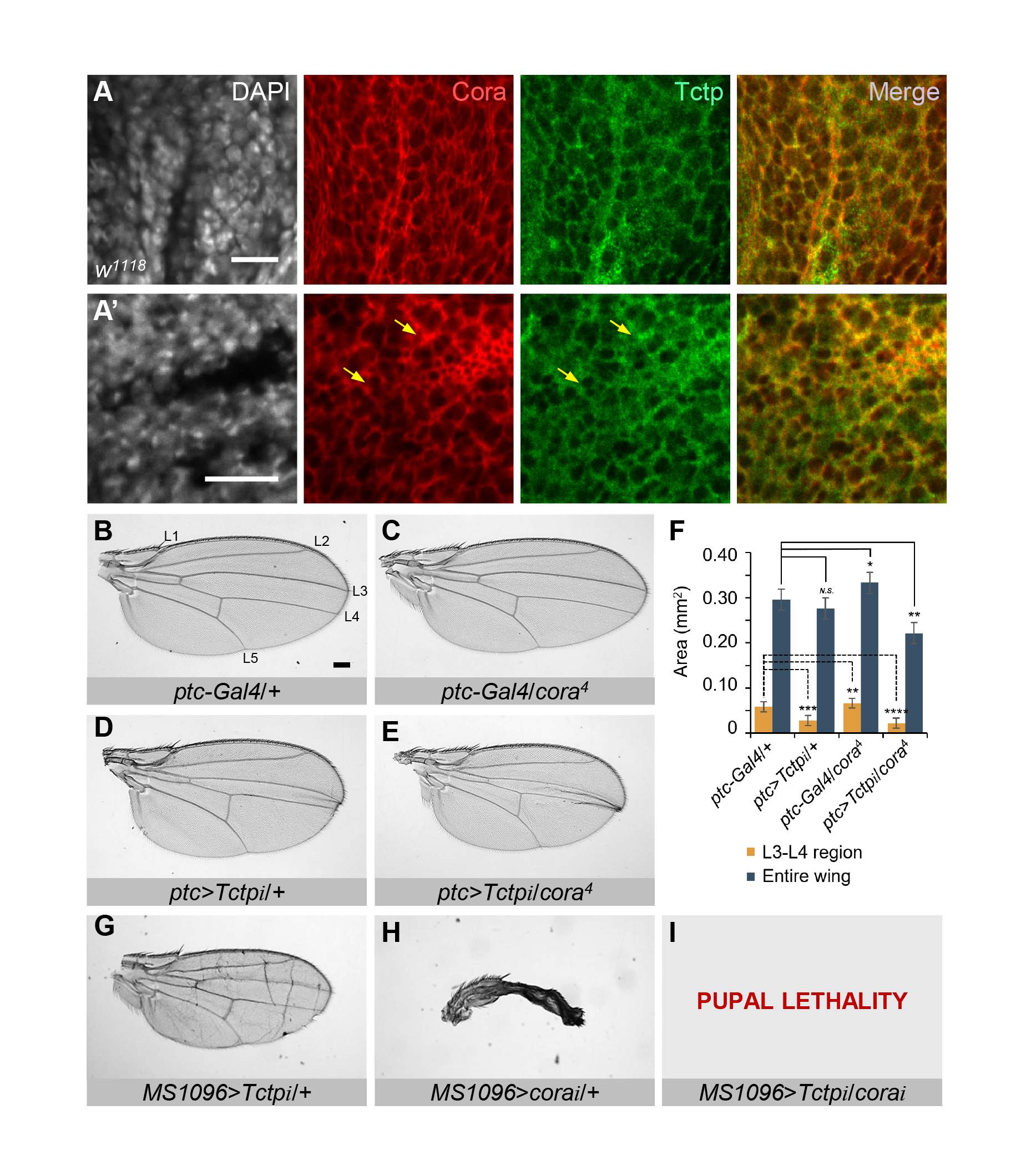

Supplement: S4 Fig — (A-A’) Immunostaining of Cora (red) and Tctp (green) in the wing disc. DAPI is shown in white. An enlarged image of a hinge region of the wing disc. Cora and Tctp overlap together in the cell membrane region. (A’) Magnification of a wing pouch region. Tctp is detected in the cytoplasm but is enriched at the membranes in a similar pattern as Cora (arrows). (B-F) Effects of RNAi driven by ptc-Gal4. Control wing with one copy of ptc-Gal4 is normal (B). cora4 /+ heterozygous wings are slightly larger than normal (C). Tctp RNAi reduces wing tissue in the ptc domain (D). Tctp RNAi phenotype is enhanced by cora4 /+ (E). Quantification of entire wing sizes and areas of ptc expression region. Blue bars indicate the entire wing area, and yellow bars shows the ptc region between L3-L4 veins (F). Error bars are s.e.m. (n = 6). N.S, not significant (P > 0.05). *P < 0.05. **P < 0.01. ***P < 0.001. ****P < 0.0001. (t-test). (G-I) Effects of RNAi driven by MS1096-Gal4. The control wing of MS1096-Gal4 is normal similar to the control wing in (B). Tctp RNAi results in the reduced and wrinkled wing (G). cora RNAi shows a very small and severely disrupted wing (H). Double knockdown of Cora and Tctp causes pupal lethality (I). White and black scale bars are 10 and 50 μm, respectively. (TIF) [file pgen.1008885.s004.tif]

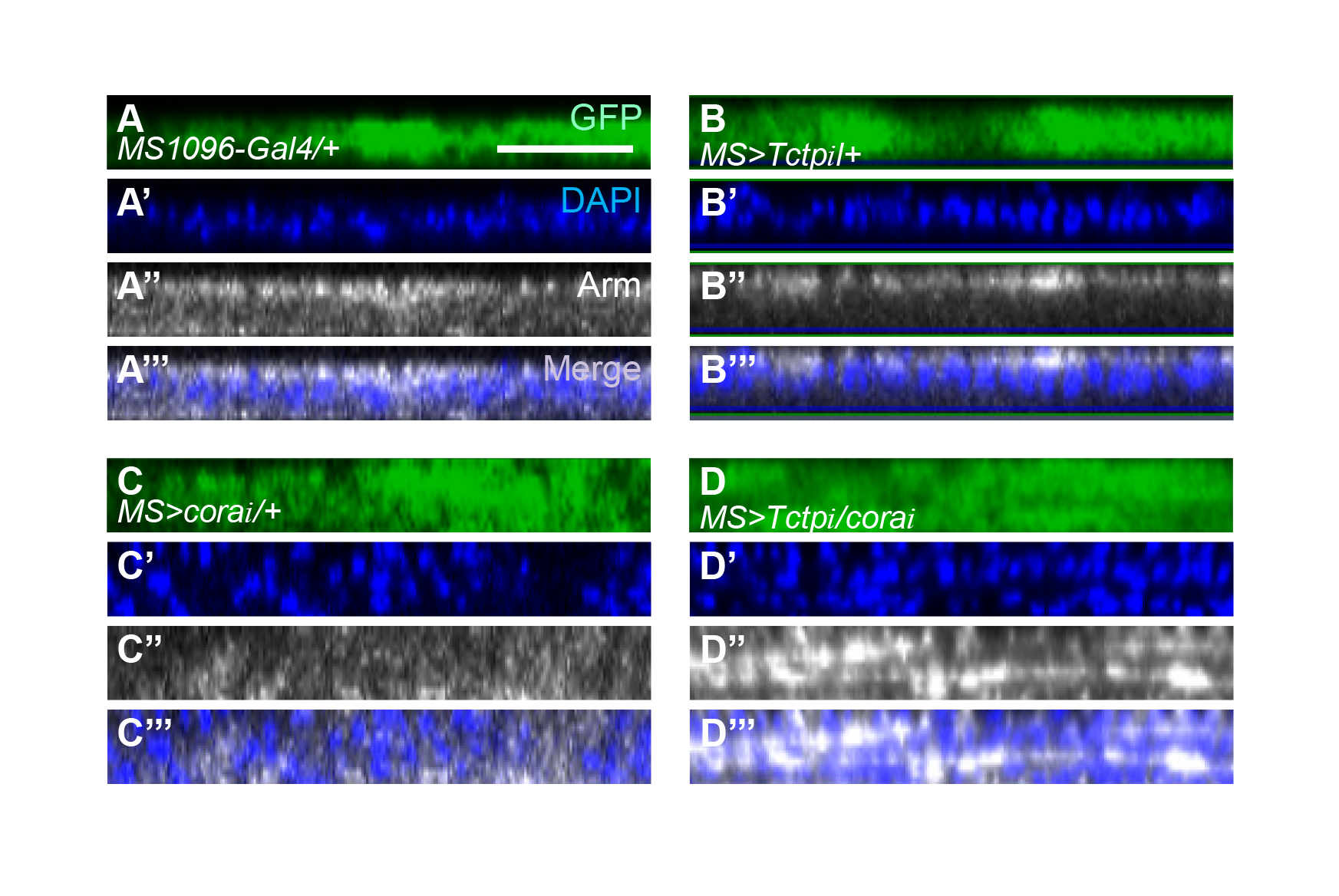

Supplement: S5 Fig — (A-D”’) Cross-section views of wing discs stained with DAPI and anti-Arm antibody. Cross-sections were made along the dorsoventral boundary of the wing disc shown as a straight line in Fig 6A. Genotypes are as indicated. RNAi was induced by MS1096-Gal4 (MS in short). Wild-type control shows staining at adherens junctions (A-A”’). Tctp RNAi shows relatively normal Arm pattern (B-B”’). cora RNAi causes a highly irregular Arm pattern and abnormal nuclei positions (C-C”’). cora/Tctp double RNAi causes abnormal positioning of nuclei and mislocalization of Arm stain to basal positions (D-D”’). Scale bar, 20 μm. (TIF) [file pgen.1008885.s005.tif]

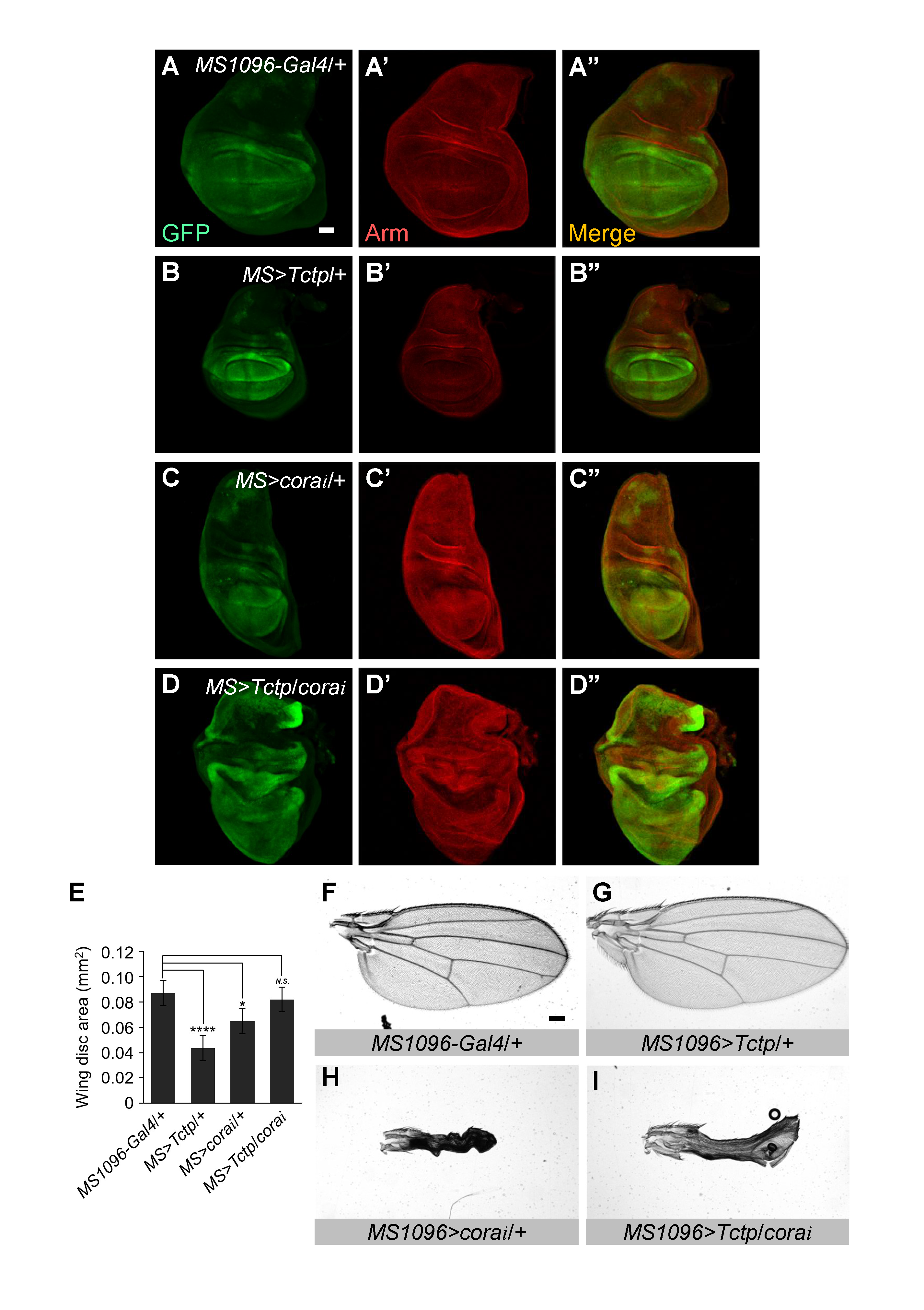

Supplement: S6 Fig — (A-D”) Wing discs of indicated genotypes were stained for GFP and Arm. RNAi was induced by MS1096-Gal4. Wild-type control (A-A”). Wing discs with Tctp overexpression are reduced in size but show normal morphology (B-B”). cora RNAi wing discs are reduced with abnormal morphology (C-C”). cora RNAi wing discs with Tctp overexpression show significant folding (D-D”). (E) Quantification of wing disc size for indicated genotypes. Error bars are s.e.m. (n ≥ 6). N.S, not significant (P > 0.05). *P < 0.05. ****P < 0.0001. (t-test). (F-I) Adult wing phenotypes in control (F), Tctp overexpression (G), cora RNAi (H) and cora RNAi with Tctp overexpression (I). (n ≥ 11). Scale bars, 50 μm. (TIF) [file pgen.1008885.s006.tif]

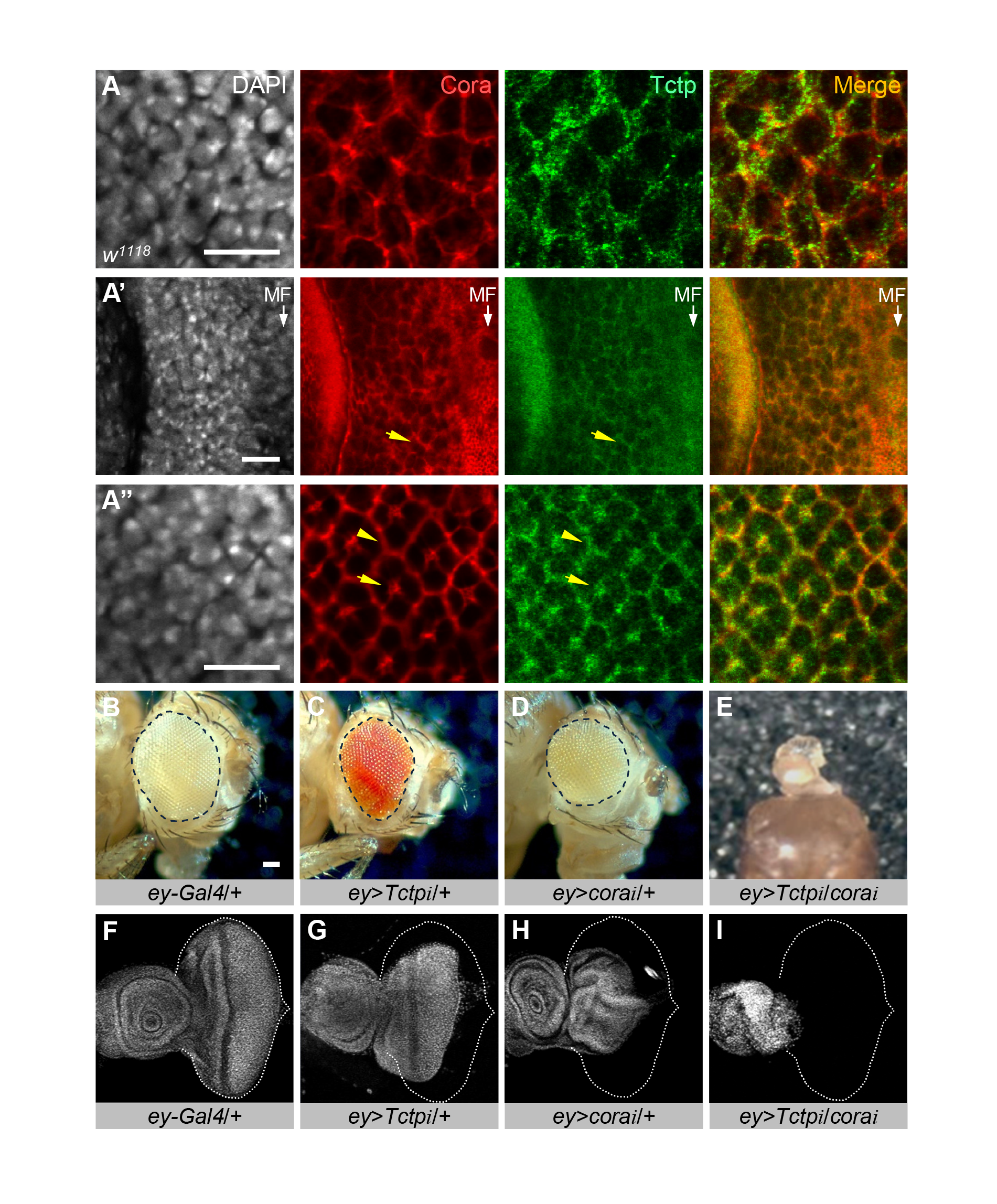

Supplement: S7 Fig — (A-A”) Wild-type eye disc stained for Cora (red) and Tctp (green). Cora and Tctp stains overlap in the peripodial membranes and eye disc proper (A’). Both Cora and Tctp are enriched in interommatidial cells (arrows) and at the center of each photoreceptor clusters where photoreceptor precursors form cell junctions. Cell nuclei are marked by DAPI (white) (A”). The position of morphogenetic furrow (MF) is indicated by white arrows. Scale bars, 10 μm. (B-E) Adult eye phenotypes. Genotypes are as indicated in each panel. ey-Gal4 control (B). The knockdown of Tctp shows a mild reduction of eye size (C). cora RNAi shows a reduced eye (D). Double knockdown of Cora and Tctp causes pupal lethality. The removal of pupal case shows loss of the eye and head structure (E). Scale bar indicates 100 μm. (F-I) 3rd instar larval eye discs stained with DAPI. ey>+ control (F). Tctp RNAi reduces the size of the eye disc (G). cora RNAi also shows small eye disc with more severity than Tctp RNAi phenotype (H). Double knockdown of Cora and Tctp shows the loss of the entire eye disc (I). Scale bar, 10 μm. (TIF) [file pgen.1008885.s007.tif]

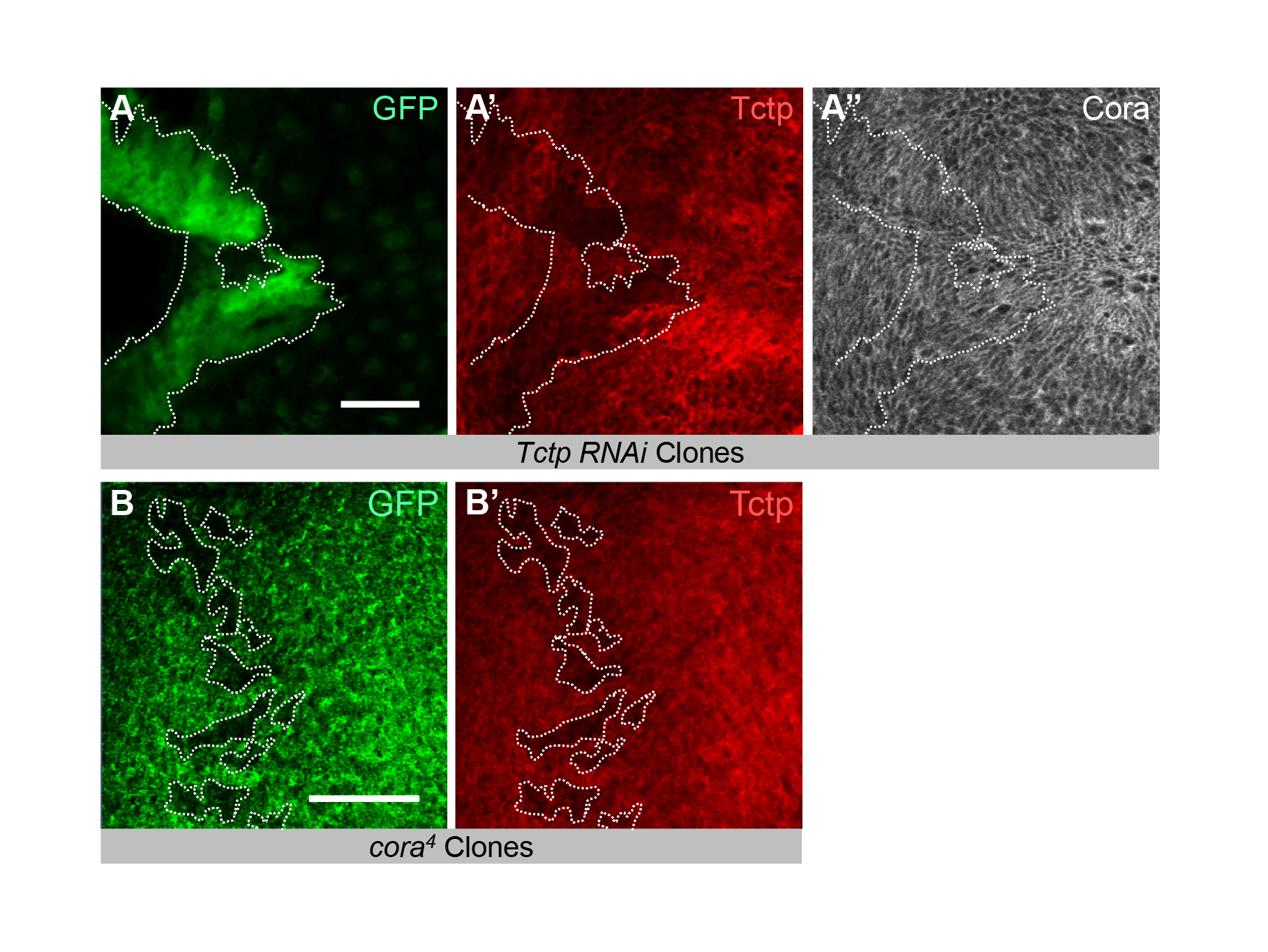

Supplement: S8 Fig — (A-A”) Tctp RNAi flp-out clones in the eye disc. RNAi clones are marked by GFP-positive cells. Tctp (red) is reduced in Tctp RNAi clones (GFP-positive), where Cora (white) shows no obvious change. (B-B’) cora4 mutant clones marked by GFP-negative cells in eye disc show reduced levels of Tctp (red). Scale bars, 20 μm. (TIF) [file pgen.1008885.s008.tif]

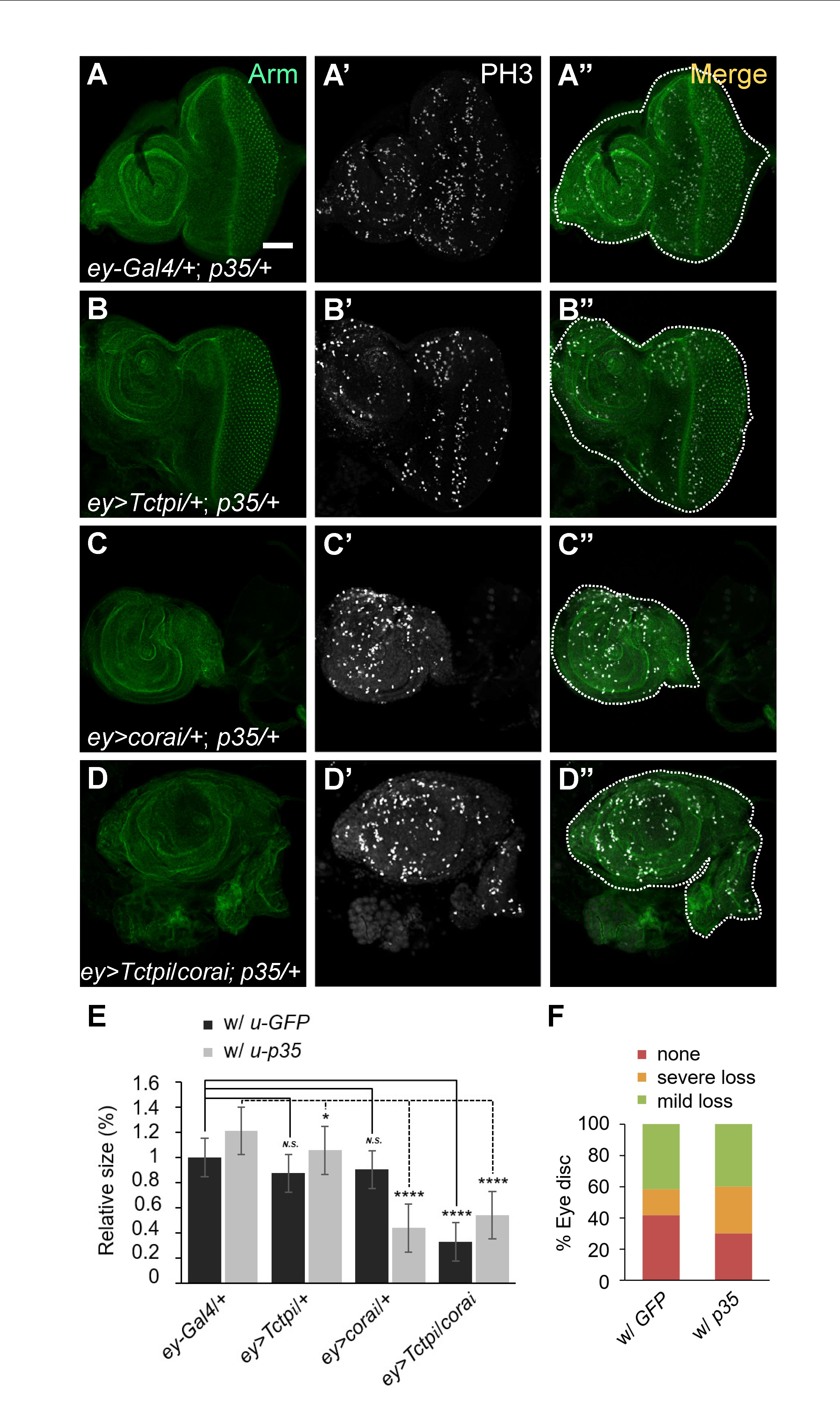

Supplement: S9 Fig — (A-D”) Eye-antenna discs were stained for Arm and PH3. Genotypes are as indicated in each panel. Eye discs in the absence of p35 are shown in S7F–S7I Fig. p35 overexpression does not affect control eye disc (A-A”). p35 overexpression weakly increases ey>Tctp RNAi eye disc (B-B”). p35 strongly reduces cora RNAi eye disc without affecting the antenna disc (C-C”). p35 overexpression causes minor recovery of eye disc from no eye-disc phenotype of cora/Tctp double RNAi (D-D”). (E) Quantification of eye disc size with and without p35 overexpression. Error bars are s.e.m. (n ≥ 6). N.S, not significant (P > 0.05). *P < 0.05. ****P < 0.0001. (t-test). (F) The percentage of eye discs showing phenotypes: none (no eye disc), severe loss (less than 25% of control eye disc size), mild loss (less than 70% of control eye disc size). (n ≥ 6). Scale bar, 50 μm. (TIF) [file pgen.1008885.s009.tif]

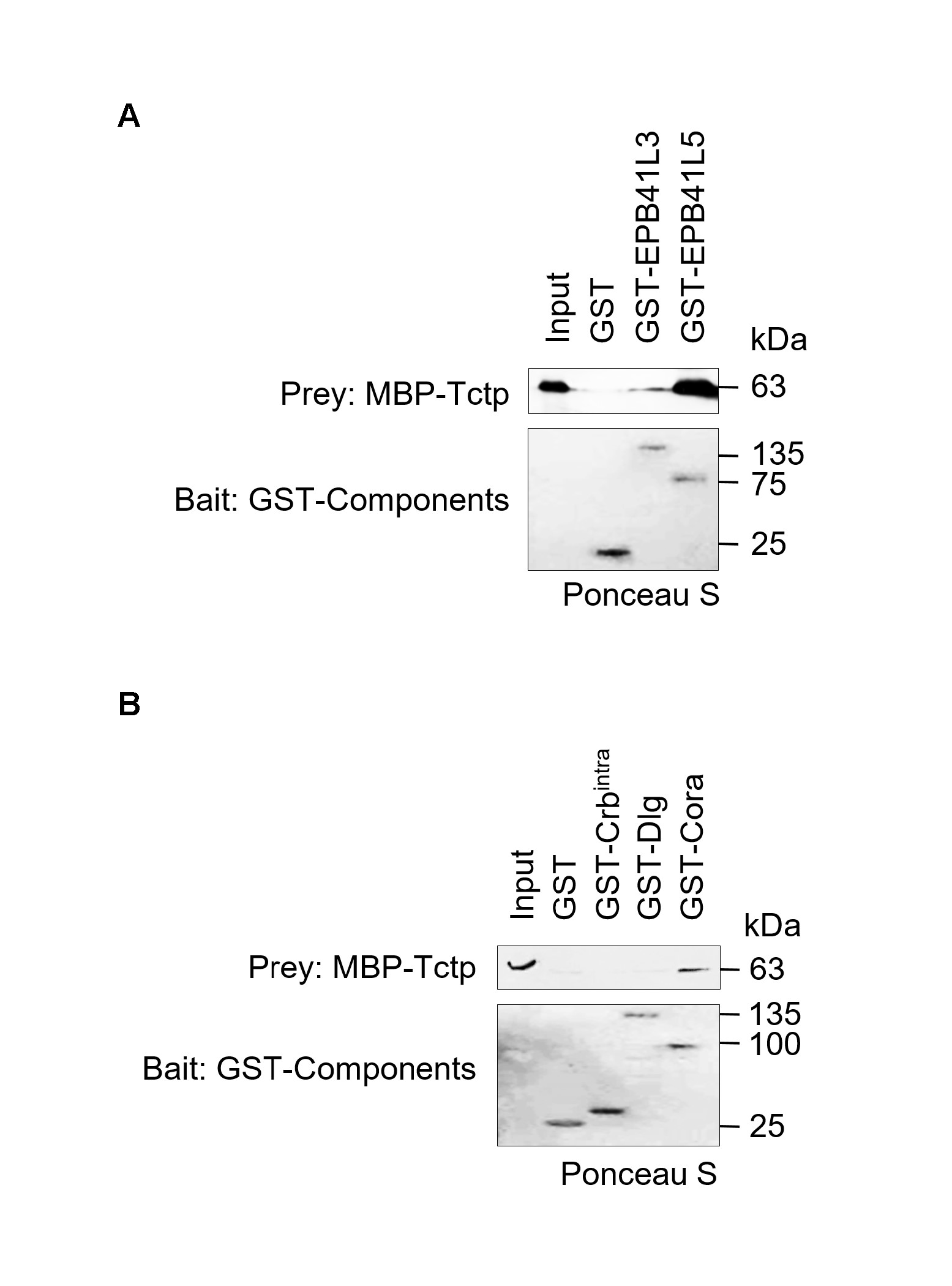

Supplement: S10 Fig — (A) GST-pulldown indicates that human TCTP binds weakly to EPB41L3 (Cora homolog) and more strongly to Yrt homolog EPB41L5. (B) GST-pulldown shows that Tctp binds to Cora but not to Crbintra and Dlg. (TIF) [file pgen.1008885.s010.tif]

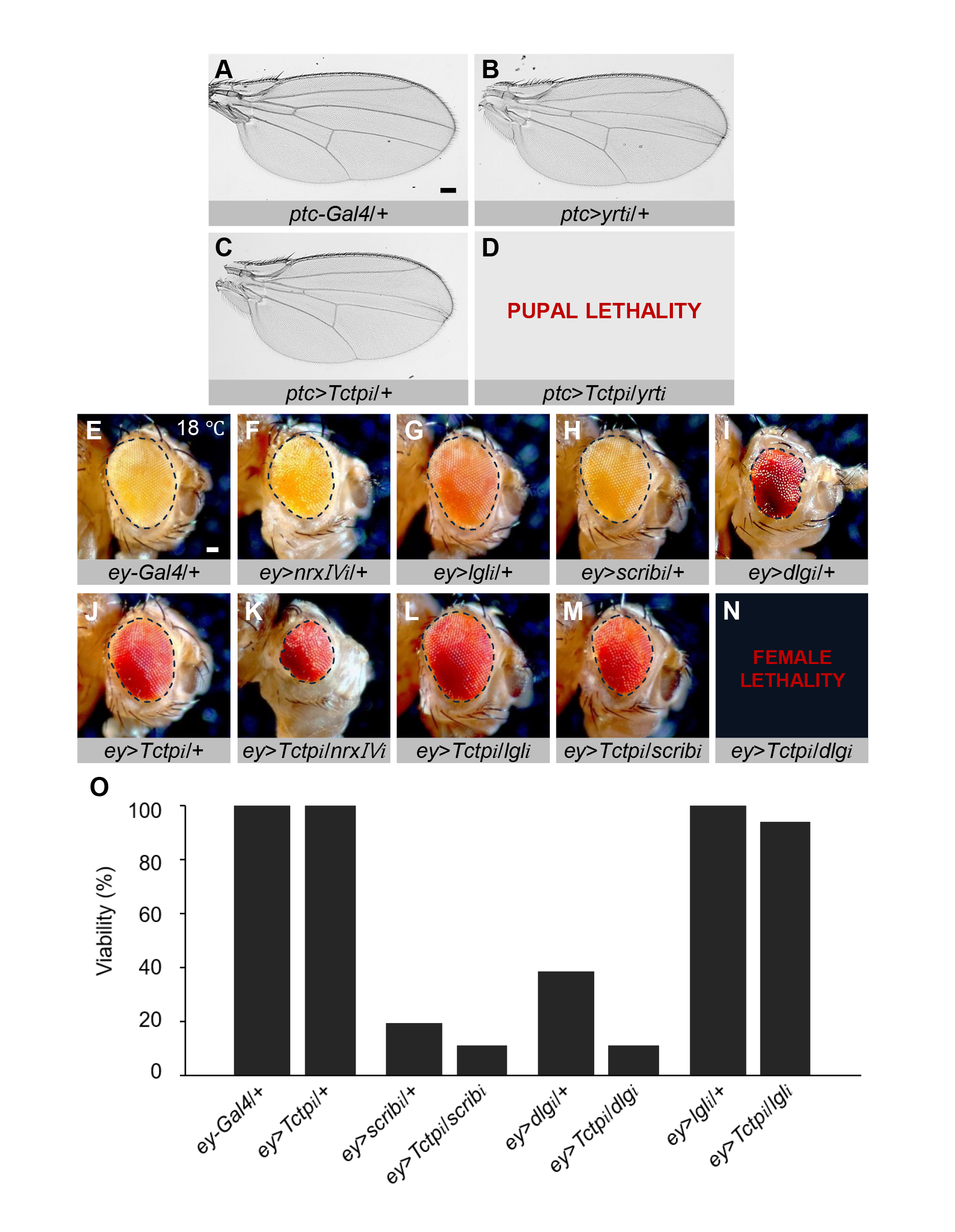

Supplement: S11 Fig — (A) ptc>+ control. (B) ptc>yrt RNAi (yrti)/+. The region between L3 and L4 vein is reduced. (C) ptc>Tctp RNAi (Tctpi)/+. The region between L3 and L4 vein is reduced. (D) ptc>Tctpi/yrti shows pupal lethality. Scale bar, 20 μm. (E-N) Genetic interaction of Tctp and other septate junction components at 18 oC. All flies are female. ey>+ control (E). Tctp RNAi causes a mild reduction in the eye (J). ey>Nrx-IV RNAi results in semi-lethality. Escapers show reduced rough eyes (F). Double knockdown of Tctp and Nrx-IV results in 94% lethality with enhanced eye phenotype (K). lgl RNAi shows normal eye (G) and has no effect on Tctp RNAi (L). scrib RNAi shows semi-lethality. Escapers show normal eye (H) but slightly enhances Tctp RNAi eye phenotype (M). dlg RNAi also causes semi-lethality, but the escapers show abnormal eye growth (I). Double RNAi for Tctp and Dlg leads to female lethality (N). (O) Quantification of relative viability for genotypes shown in E, G-J, L-N. The same number of males and females was used for crosses, and viability was relative to ey>+ control. Scale bar, 100 μm. (n ≥ 18). (TIF) [file pgen.1008885.s011.tif]
